# Supplementary material for: Building Damage-Resilient Dominating Sets in Complex Networks against Random and Targeted Attacks
Source: Sci Rep. 2015 Feb 9;5:8321. doi: 10.1038/srep08321 (PMC4321165; doi:10.1038/srep08321)
Supplement: Supplementary Information [file srep08321-s3.pdf]

Building Damage-Resilient Dominating Sets in Complex Networks against Random and Targeted Attacks
Supplementary Information

F. Molnár Jr.[1,2,*], N. Derzsy[1,2], B. K. Szymanski[2,3], G. Korniss[1,2]

December 3, 2014

1 Department of Physics, Rensselaer Polytechnic Institute, 110 8th Street, Troy, NY, 12180-3590 USA
2 Social Cognitive Networks Academic Research Center, Rensselaer Polytechnic Institute, 110 8th Street, Troy, NY, 12180-3590 USA
3 Department of Computer Science, Rensselaer Polytechnic Institute, 110 8th Street, Troy, NY, 12180-3590 USA

Supplementary Movie 1

Declining stability of the minimum dominating set (MDS) in a scale-free network, while being subjected to random node removal. Parameters: $N = 1000$, $\langle k \rangle = 5$, $\gamma = 2.5$. Available online.

Supplementary Movie 2

Declining stability of the minimum dominating set (MDS) in a scale-free network, while being subjected to removal of nodes in degree-ranked order. Parameters: $N = 1000$, $\langle k \rangle = 5$, $\gamma = 2.5$. Available online.

Supplementary Note 1
Algorithm for Finding a Flexible-Redundancy Dominating Set (frDS)

The algorithm for finding a flexible-redundancy dominating set (frDS) is based on greedy search. At each step we add one node to the dominating set, that helps the maximum number of nodes to advance toward their required domination goals. There are several variables that we must define and track for each node.

First, we define the domination requirement $r(i)$ as the number of required dominators for node i among its closed neighbors. This value is calculated and assigned randomly for each node before the search begins. The requirement is either $\lfloor r \rfloor$ or $\lceil r \rceil$ (where r is the global requirement for the entire network), the probability for the latter is exactly the fractional part of r (that is, $r - \lfloor r \rfloor$). Note, that $r(i)$ can be zero if $r < 1$, and it is also possible that $r > d(i)+1$ (where $d(i)$ is the degree of node i), in which case all nodes in the closed neighborhood are required to be in the dominating set.

Second, we define $score(i)$ as the current number of dominators of node i at any given step. Initially, $score(i) = 0$ for every node, and it increases by one in the closed neighborhood of the selected node.

Finally, we track the dominating $potential(i)$ of node i, which counts how many nodes in the closed neighborhood of i have not yet reached their domination requirement. Specifically,

*E-mail: molnaf@rpi.edu

$potential(i) = \sum_{j \in N^+(i)} I[score(j) < r(j)]$, where $N^+(i)$ is the closed neighborhood of i and $I[x]$ is an indicator function that returns 1 if x is true and 0 else. In other words, the potential is the number of nodes in the closed neighborhood that can be advanced toward their goal by selecting i as the next dominator. The greedy search is based on this quantity: at every step we select a node with maximum potential (with random tie-breaking among the candidates).

The key to implementing the algorithm with optimal time complexity is the use of an efficient data structure for maintaining a list of nodes sorted by their potentials. Note that the potential is an integer value between 0 and $N+1$, therefore we can use bucket-sort for initial sorting. We assign one bucket for each possible potential value, and we implement each bucket by a hashed set. This way we can add or remove a node from any bucket in $O(1)$ step, therefore we can perform the initial sorting in $O(N)$ and maintain sortedness in $O(1)$ step after any single change in a node's potential.

The time complexity of the algorithm can be found by analyzing the changes in scores and potentials of nodes. The initial calculation of potentials requires a loop over all nodes' all neighbors. Assuming we can enumerate the neighbors of node i in $d(i)$ steps, this calculation takes $\sum_{i \in V(G)} 1 + d(i) = 2E + N = O(E)$ steps. Then in the main loop one node is selected at every step, which increases the score of the selected node and its neighbors by one. In principle, the scores could increase until all nodes are selected (e.g., when $r > N$), therefore again all nodes' all neighbors are processed, taking $O(E)$ steps. However, during this procedure, there are additional steps for updating the node potentials. Some (usually all) nodes will reach their predefined requirement at one point or another, after which the dominating potentials change. We count these changes as follows. Initially, all nodes can increase all their neighbors' score toward their requirement (including the nodes themselves), therefore the initial sum of potentials is $\sum_{i \in V(G)} 1 + d(i) = 2E + N$, or less, if some nodes have zero requirement. The potential of a node can either be reduced by one if a neighbor reaches its requirement (and thus that neighbor can no longer be advanced to its goal by the current node), or it becomes zero by definition if the node is actually selected. At most, there are $2E + N = O(E)$ changes (reductions) of potentials, each computed in $O(1)$ time (maintaining sortedness of nodes after each change), therefore during the procedure there are at most $O(E)$ additional steps for updating node potentials. This means the entire algorithm runs in $O(E)$ steps. Note that in sparse networks, $O(E) = O(N)$.

Algorithm S1 Find an frDS

```
procedure FRDS(G: graph, r: domination redundancy)
    finished ← 0
    for all i ∈ V(G) do                                   ▷ initialization of score, r, and potential
        score(i) ← 0
        potential(i) ← 0
        if Random(0,1) < r − ⌊r⌋ then
            r(i) ← ⌈r⌉
        else
            r(i) ← ⌊r⌋
        end if
        if score(i) ≥ r(i) then
            finished ← finished + 1
        end if
    end for
    for all i ∈ V(G) do                                   ▷ initial calculation of potentials
        for all j : (i,j) ∈ E(G) do
            if score(j) < r(j) then
                potential(i) ← potential(i) + 1
            end if
        end for
        if score(i) < goal(i) then
            potential(i) ← potential(i) + 1
        end if
    end for
    while finished < |V(G)| ∧ max(potential) > 0 do        ▷ main loop
        k := random node with maximum potential           ▷ greedy step
        Add k to Dominating Set                           ▷ construct the output
        score(k) ← score(k) + 1                           ▷ count self-domination
        potential(k) ← 0                                  ▷ remove k from further consideration
        change ← score(k) = r(k)                          ▷ requirement of k reached in this iteration?
        if change then
            finished ← finished + 1
        end if
        for all j : (j,k) ∈ E(G) do                       ▷ update neighbors of k
            if change then                                ▷ neighbors cannot increase score(k) any more
                potential(j) ← max(0, potential(j) − 1)
            end if
            score(j) ← score(j) + 1                       ▷ k adds domination score to all its neighbors
            if score(j) = r(j) then                       ▷ requirement reached for the neighbor?
                finished ← finished + 1
                potential(j) ← max(0, potential(j) − 1)
                for all x : (x,j) ∈ E(G) do               ▷ update potentials of second neighbors
                    if x ≠ k then                         ▷ skip when second neighbor is k
                        potential(x) ← max(0, potential(x) − 1)
                    end if
                end for
            end if
        end for
    end while
end procedure
```

Supplementary Note 2
Algorithm for Finding a Flexible-Cost Dominating Set (fcDS)

The fcDS algorithm is also a form of greedy search, since it builds the dominating set by selecting one node at a time with maximum potential, similarly to the frDS algorithm. However, in this method the potential is calculated from the changes in probability of losing all dominators for the nodes in the neighborhood of the given node.

First, we define $strength(i)$ for each node i as an input ($0 < strength(i) < 1$), which defines the probability of not losing node i after the anticipated damage:

$$strength(i) := \Pr(i \text{ is not lost}). \tag{1}$$

We also keep a record of $instability(i)$ for each node i, which is defined as the probability of losing all dominators after the damage has occured:

$$instability(i) = \prod_{j \in \mathrm{DS} \cap N^+(i)} 1 - strength(j). \tag{2}$$

Initially, $instability(i) = 1.0$ for all i. The $potential(i)$ of node i, which is used in the greedy node selection, is calculated as the sum of the changes in instabilities over the closed neighborhood of node i, if i was selected:

$$\begin{aligned} potential(i) &= - \sum_{j \in N^+(i)} instability(j) \Pr(i \text{ is lost}) - instability(j) \\ &= - \sum_{j \in N^+(i)} instability(j) \left[\Pr(i \text{ is lost}) - 1\right] \\ &= \sum_{j \in N^+(i)} instability(j) strength(i). \end{aligned} \tag{3}$$

Note, that the negative sign is manually inserted to make the potential a positive value, for practical reasons. Without it, the change in instabilities would be negative, because by each node selection the stability always increases.

With the definition above, we select a node with maximum potential at each greedy step. After the node has been selected and added to the dominating set, the instabilities in the closed neighborhood, and the potentials for all nodes in the second neighborhood of the selected node must be recalculated, and the nodes must be sorted again based on the new potentials. Since the potentials are non-integer values (and cannot be mapped to integers) we can only use comparative sorting, where it takes $O(\log N)$ steps to find the new place for each node in the list. With a simple approximation for sparse networks, a node in a network with average degree d will have $O(d^2)$ nodes in its second neighborhood, therefore the selection of each dominator involves $O(d^2 \log N)$ steps.

Algorithm S2 Find an fcDS

```
procedure fcDS(G: graph, strength: array, c: number of nodes to select)
    for all i ∈ V(G) do                              ▷ initialization of instability and potential
        instability(i) ← 1.0
        potential(i) ← (degree(i) + 1)(1 − strength(i))
    end for
    for a ← 1 ... c do                               ▷ a simply counts the output
        k ← random node with maximum potential       ▷ greedy selection
        Add k to Dominating Set                      ▷ construct the output
        S ← ∅                                        ▷ set of nodes whose potential must be updated
        instability(k) ← instability(k)(1 − strength(k))    ▷ update self instability
        for all j : (k, j) ∈ E(G) do
            instability(j) ← instability(j)(1 − strength(k)   ▷ update instability of neighbors
            S ← S ∪ {j}                              ▷ request potential update for j
            for all i : (j, i) ∈ E(G) do
                S ← S ∪ {i}                          ▷ request potential update for second neighbors
            end for
        end for
        for all i ∈ S do                             ▷ update potentials
            potential(i) ← 0
            if i ∉ Dominating Set then
                for all j : (i, j) ∈ E(G) do
                    potential(i) ← potential(i) + instability(j)strength(i)
                end for
                potential(i) ← potential(i) + instability(i)strength(i)
            end if
        end for
    end for
end procedure
```

Supplementary Note 3
Measuring and Controlling Assortativity

Assortativity of a network, defined loosely, is the tendency that nodes of similar degrees are connected to each other. We measure assortativity of a network using Spearman's ρ [1], which has been shown recently [2] to be a much more accurate measure of assortativity than Newman's assortativity coefficient [3, 4]. Spearman's ρ values have a range of $(-1, 1)$, ranging from completely disassortative to completely assortative. Note, that a network with $\rho = 0$ is called an uncorrelated network.

In our recent work [5] we have proposed a method to control the assortativity of a network using a Markov-chain of double-edge swaps [6], guided by biased edge-swap acceptance probabilities. These swaps preserve the degree sequence of a network, but change its assortativity. We provide here an overview of this method again for the reader's convenience.

In our method, we look for randomly (uniformly) selected pairs of edges that have four distinct nodes and no common incident edges. These edge pairs allow for a double-edge swap. However, instead of accepting all swaps, we only accept them with the following probability:

$$\Pr(\text{accept}) = \begin{cases} p & \text{if } p > 0 \text{ and the swap makes the network more assortative} \\ -p & \text{if } p < 0 \text{ and the swap makes the network more dissortative} \\ 1 - |p| & \text{else,} \end{cases} \tag{4}$$

where $-1 < p < 1$ is a parameter that controls the acceptance ratio of assortative and dissortative swaps. A swap is classified as assortative or dissortative if it increases or decreases the assortativity coefficient [3, 4] of the network, which can be evaluated very quickly.

Using our guided edge-mixing, we can reach a wide range of ρ values for any given network; however, determining the correct p value for a desired ρ is nontrivial. Due to the random nature of the mixing procedure, the resulting value of ρ is a random variable. The mean of ρ monotonically increases as p increases, allowing us to use a randomized bisection search to find the needed p for a desired ρ.

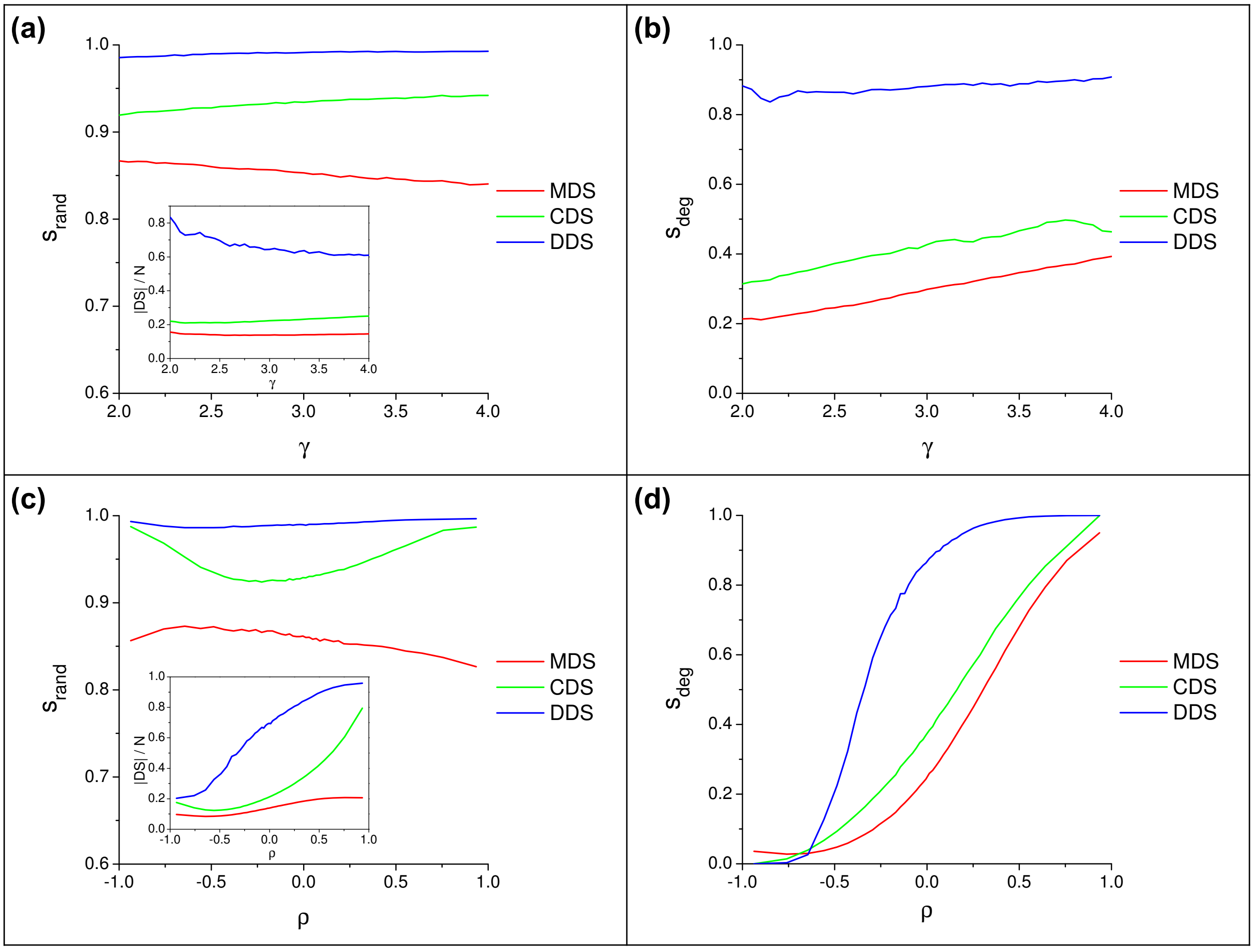

Supplementary Figure S1: Stability of dominating sets vs. power-law degree exponent γ and Spearman's ρ assortativity measure. (a) and (c) present random node removal, (b) and (d) show degree-ranked node removal. The insets illustrate the sizes of the corresponding dominating sets. In (a) and (b): $\rho = 0.0$; in (c) and (d): $\gamma = 2.5$. Common parameters: $N = 5000$, $\langle k \rangle = 8$, $f = 0.3$. Results are averaged over 200 network samples.

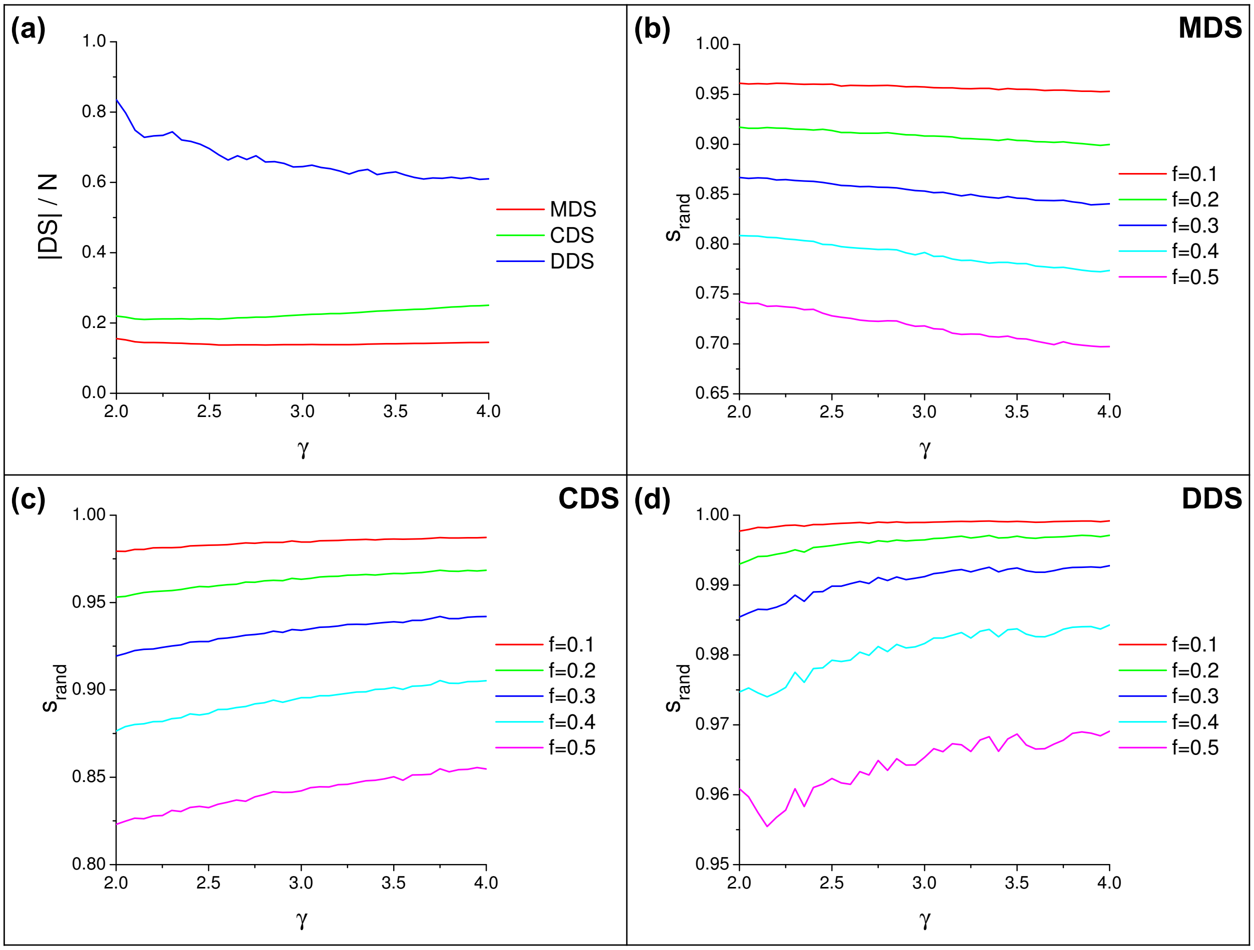

Supplementary Figure S2: Comparison of size and stability of dominating sets vs. power-law degree exponent, at various fractions of random node removal. Synthetic networks, $N = 5000$, $\langle k \rangle = 8$, $\rho = 0.0$. Results are averaged over 200 network samples.

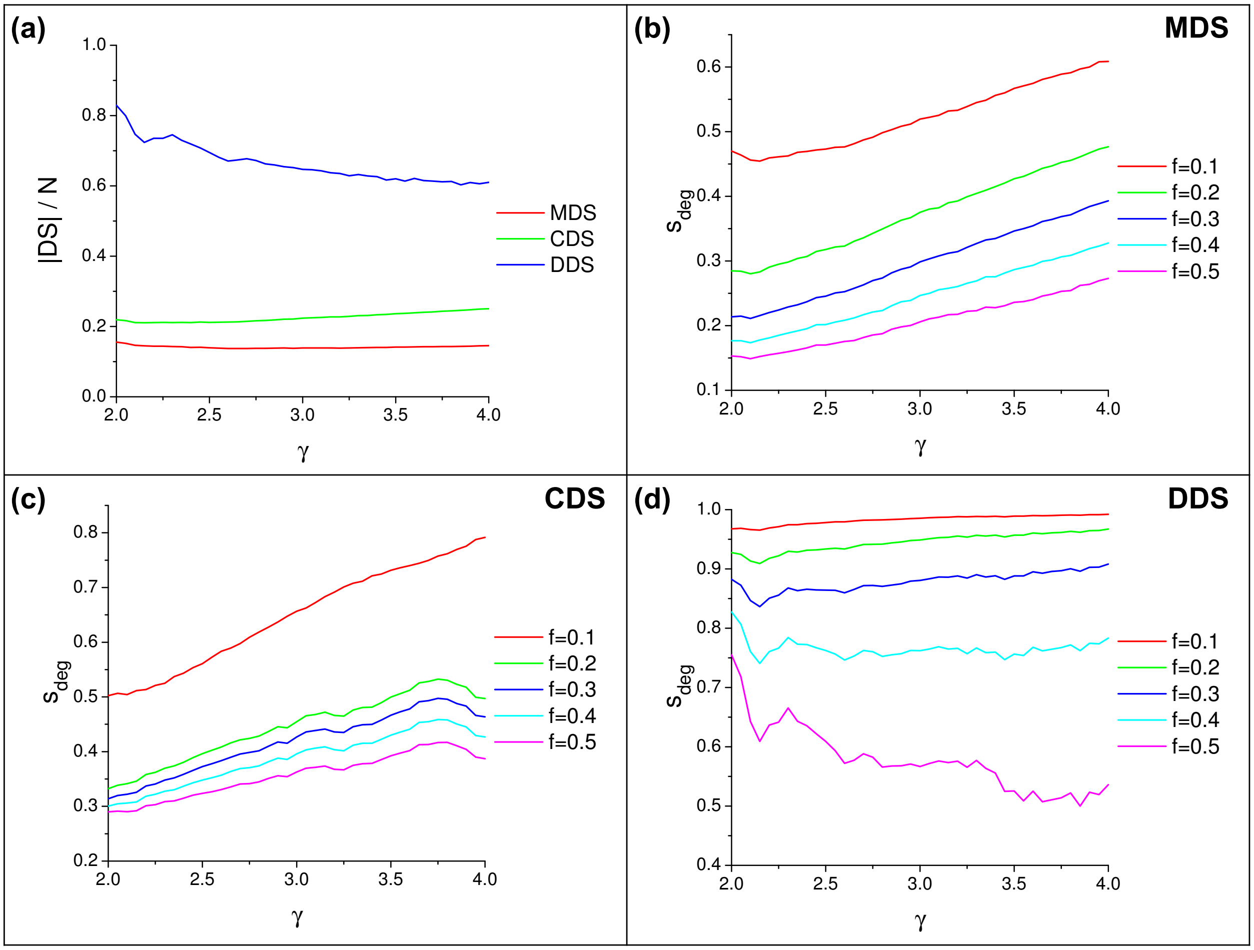

Supplementary Figure S3: Comparison of size and stability of dominating sets vs. power-law degree exponent at various fractions of degree ranked node removal. Synthetic networks, $N = 5000$, $\langle k \rangle = 8$, $\rho = 0.0$. Results are averaged over 200 network samples.

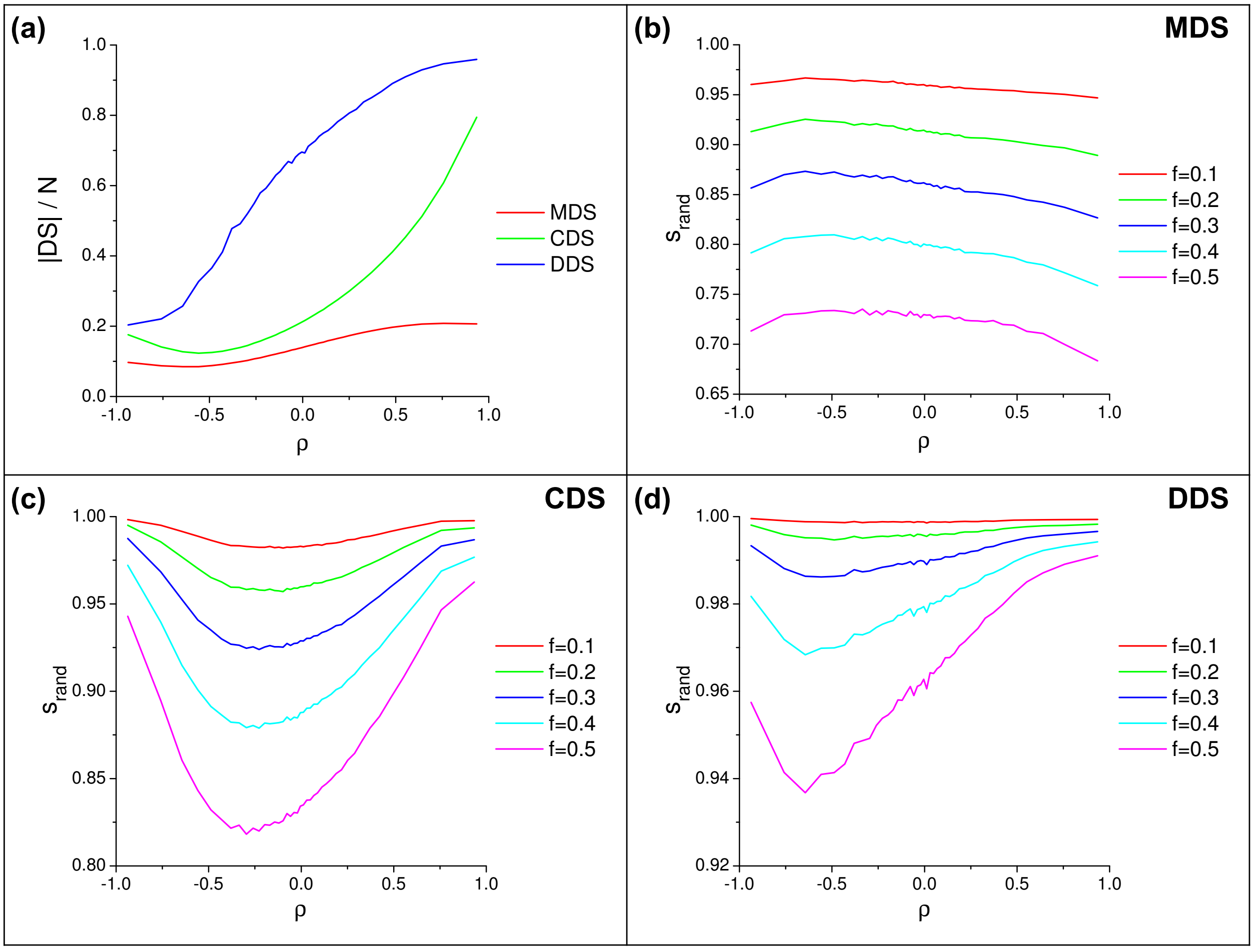

Supplementary Figure S4: Comparison of size and stability dominating sets vs. assortativity at various fractions of random node removal. Synthetic networks, $N = 5000$, $\langle k \rangle = 8$, $\gamma = 2.5$. Results are averaged over 200 network samples.

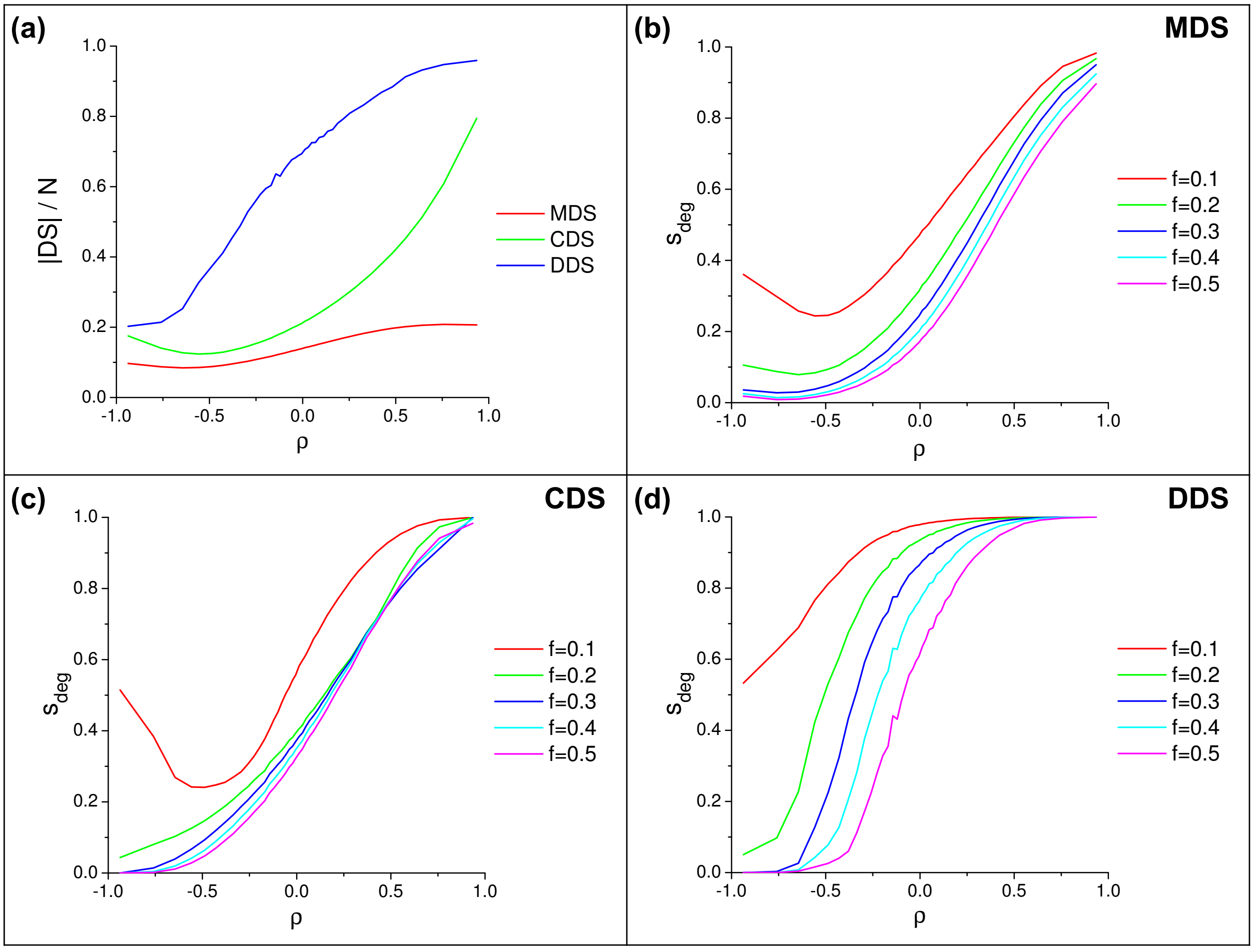

Supplementary Figure S5: Comparison of size and stability dominating sets vs. assortativity at various fractions of degree-ranked node removal. Synthetic networks, $N = 5000$, $\langle k \rangle = 8$, $\gamma = 2.5$. Results are averaged over 200 network samples.

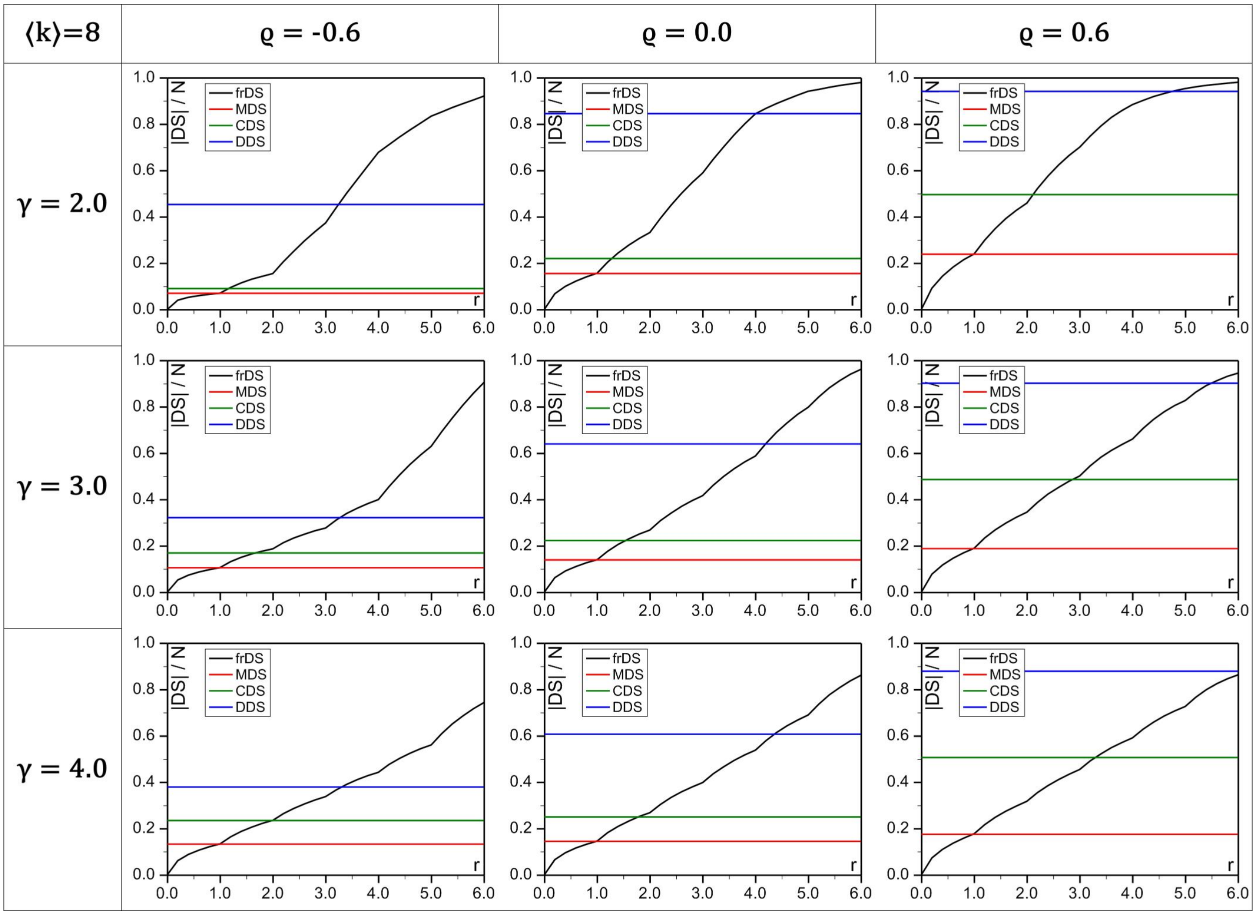

Supplementary Figure S6: Size of frDS as a function of domination redundancy, at various power-law degree exponents and Spearman's ρ values, in synthetic networks with $N = 5000$. The sizes of MDS, CDS, and DDS are shown for comparison.

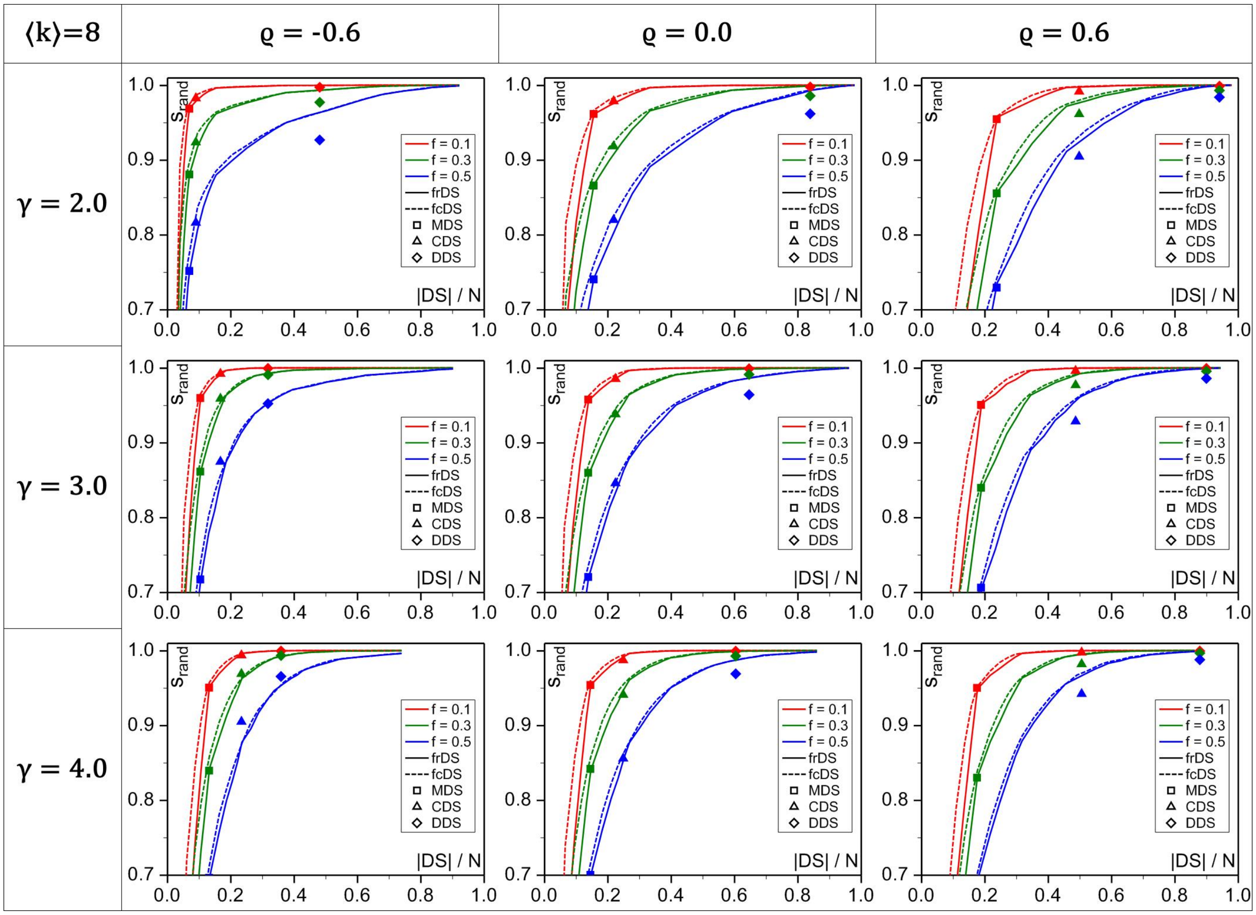

Supplementary Figure S7: Stability of frDS (solid lines) and fcDS (dotted lines) against random damage at given damage fraction f, as a function of dominating set size (cost), at various power-law degree exponents and Spearman's ρ values, in synthetic networks with $N = 5000$. The stabilities of MDS, CDS, and DDS are shown for comparison at their corresponding set sizes. Black legend symbols refer to the shape only, colors refer to damage fractions.

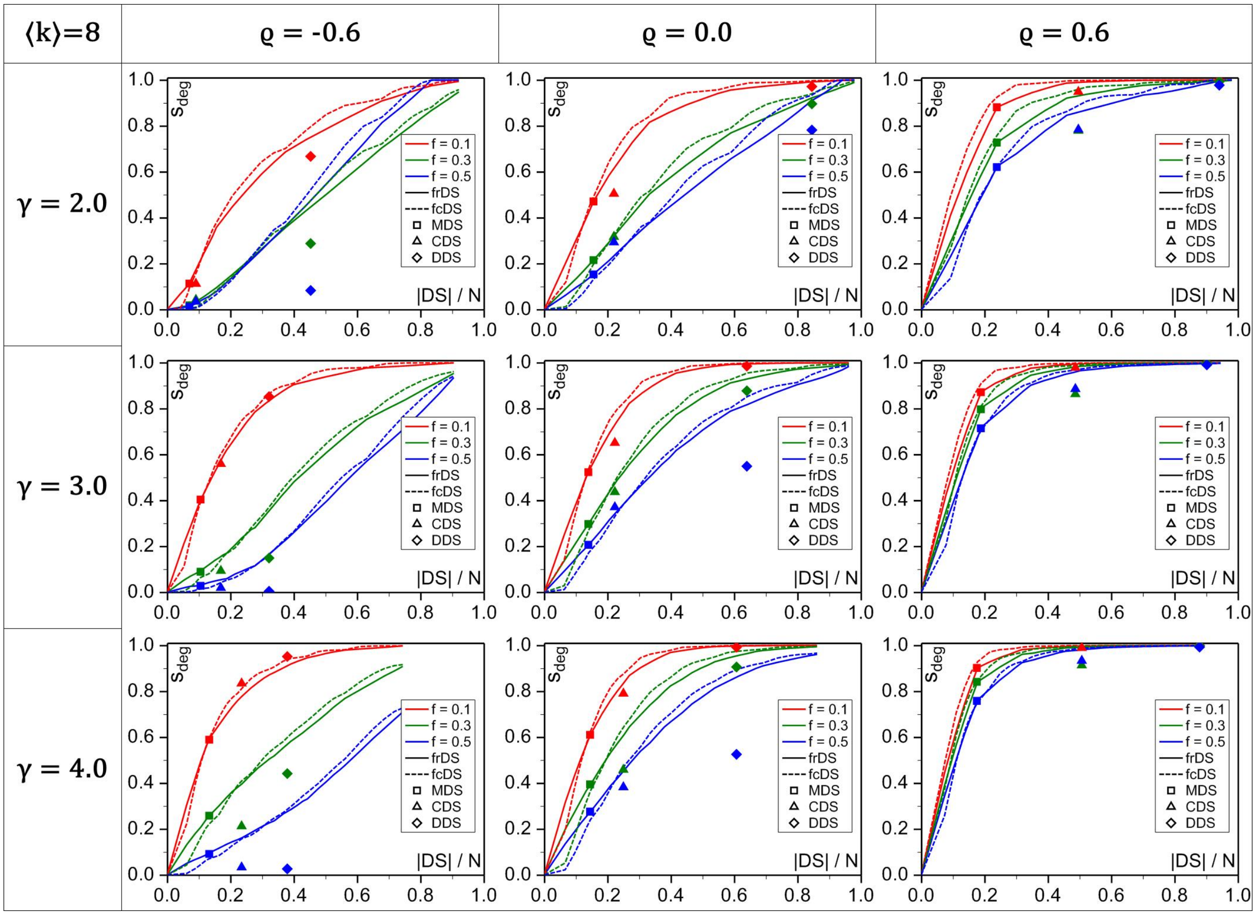

Supplementary Figure S8: Stability of frDS (solid lines) and fcDS (dotted lines) against targeted attack at given damage fraction f, as a function of dominating set size (cost), at various power-law degree exponents and Spearman's ρ values, in synthetic networks with $N = 5000$. The stabilities of MDS, CDS, and DDS are shown for comparison at their corresponding set sizes. Black legend symbols refer to the shape only, colors refer to damage fractions.

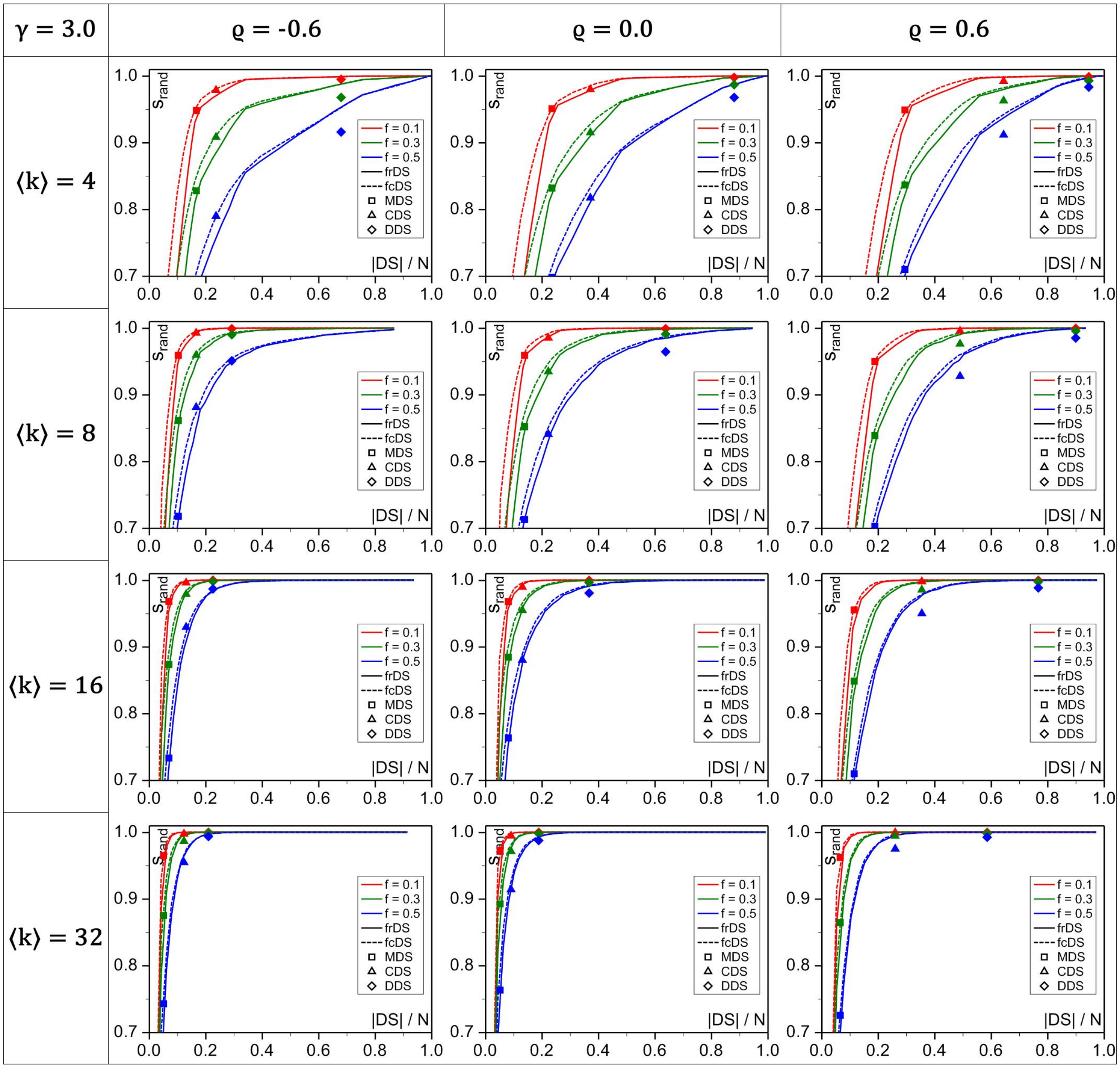

Supplementary Figure S9: Stability of frDS (solid lines) and fcDS (dotted lines) against random damage at given damage fraction f, as a function of dominating set size (cost), at various average degree and Spearman's ρ values, in synthetic networks with $N = 5000$. The stabilities of MDS, CDS, and DDS are shown for comparison at their corresponding set sizes. Black legend symbols refer to the shape only, colors refer to damage fractions.

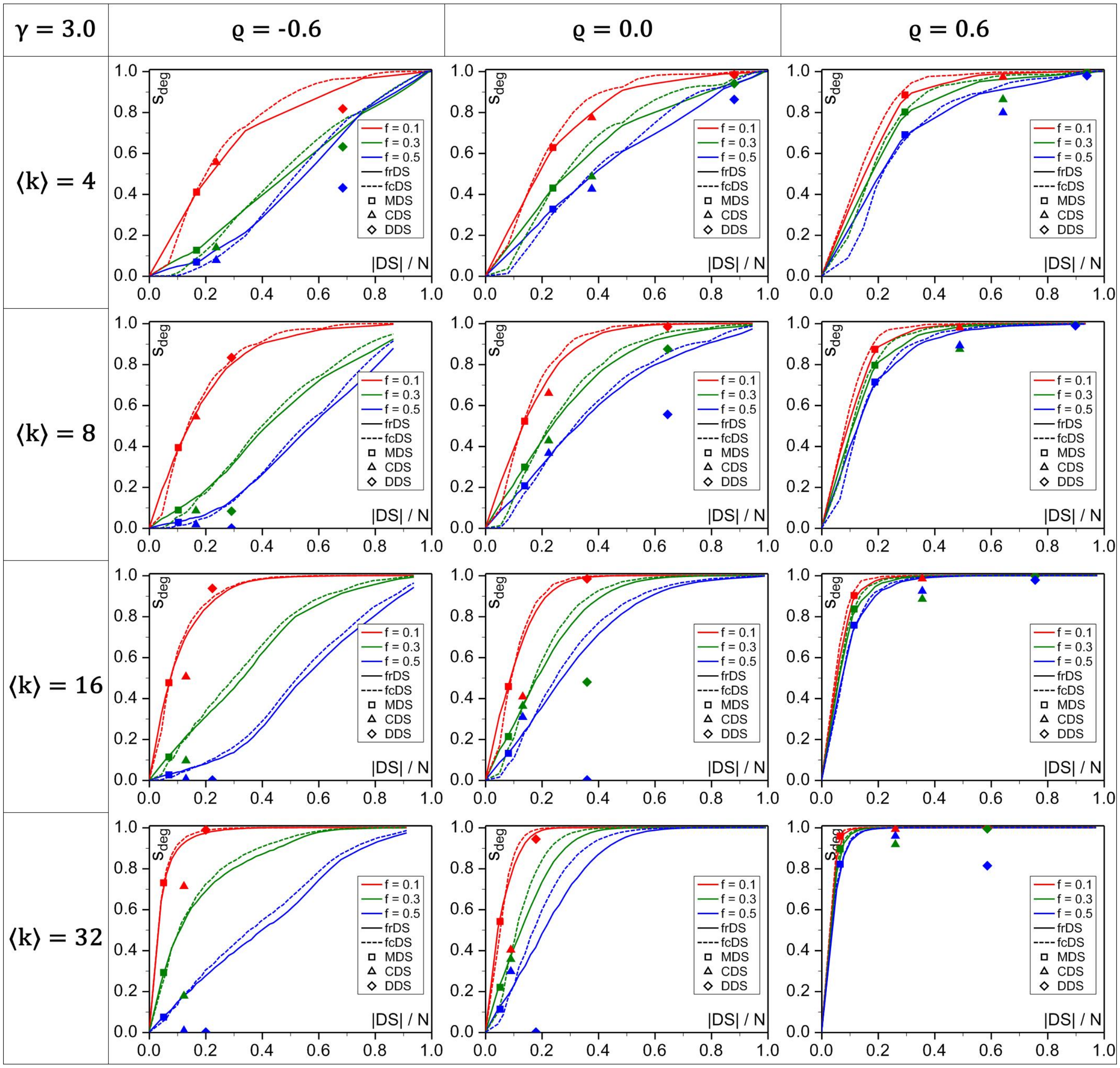

Supplementary Figure S10: Stability of frDS (solid lines) and fcDS (dotted lines) against targeted attack at given damage fraction f, as a function of dominating set size (cost), at various average degree and Spearman's ρ values, in synthetic networks with $N = 5000$. The stabilities of MDS, CDS, and DDS are shown for comparison at their corresponding set sizes. Black legend symbols refer to the shape only, colors refer to damage fractions.

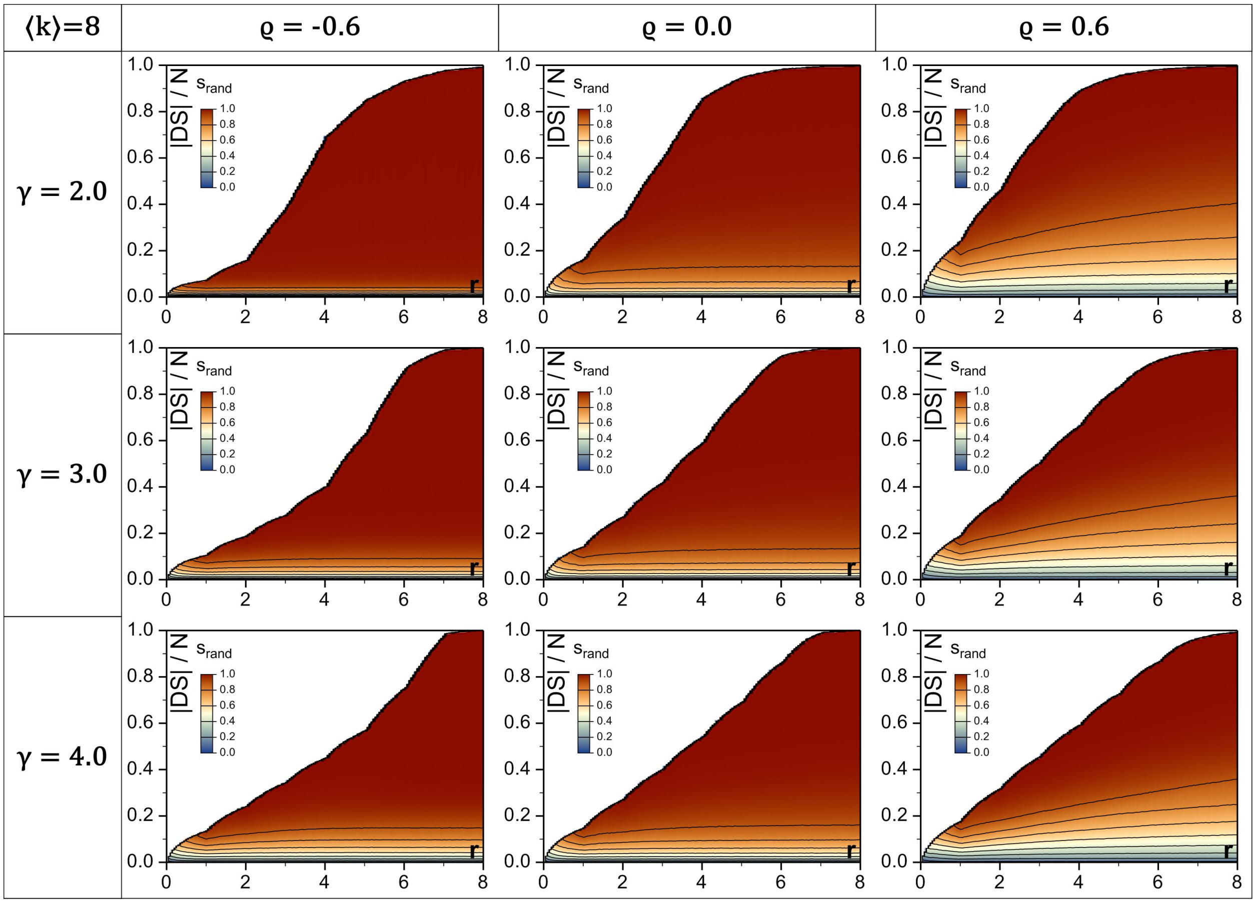

Supplementary Figure S11: Stability of partial frDS against random damage, as a function of redundancy level and dominating set size, at various power-law degree exponents and Spearman's ρ values. Synthetic networks, $N = 5000$, damage fraction $f = 0.1$.

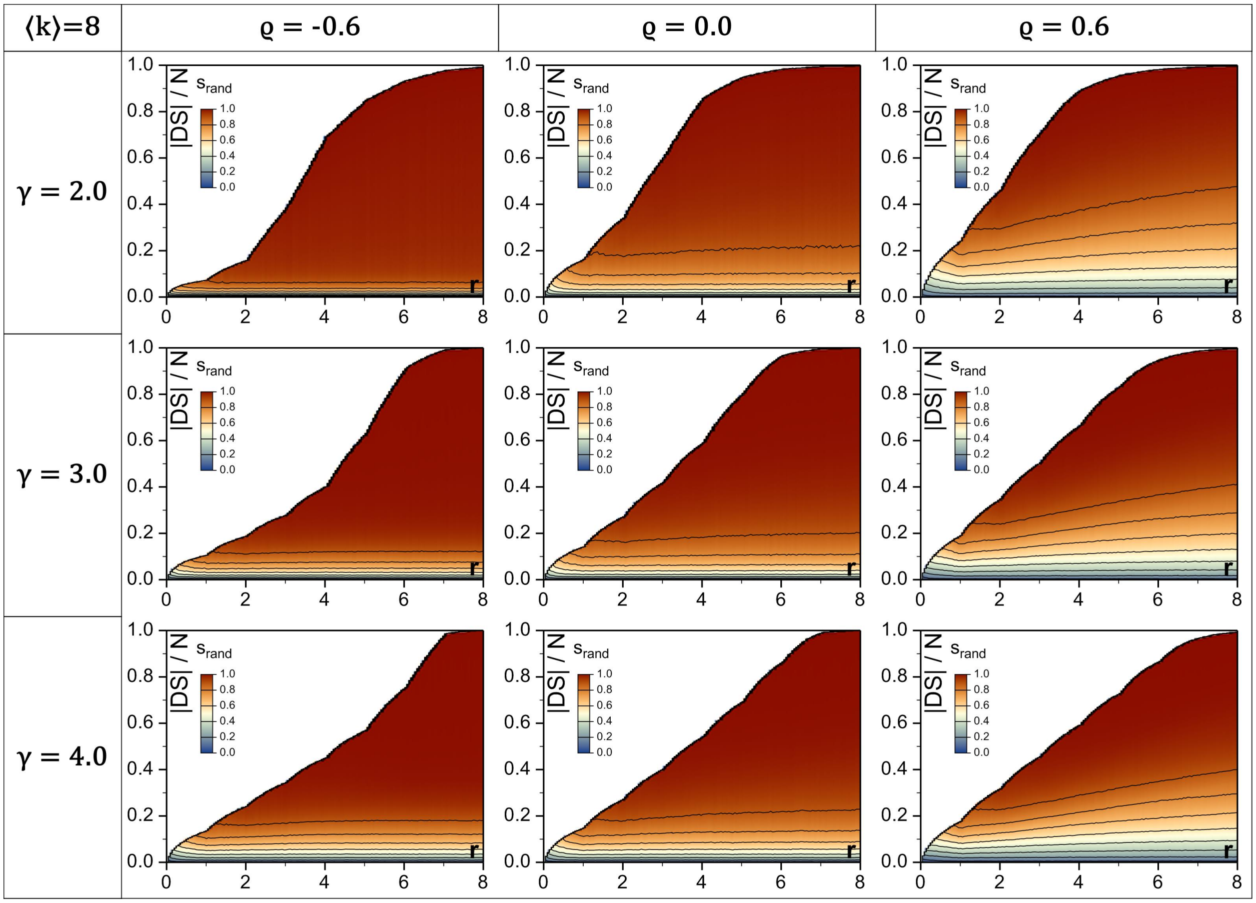

Supplementary Figure S12: Stability of partial frDS against random damage, as a function of redundancy level and dominating set size, at various power-law degree exponents and Spearman's ρ values. Synthetic networks, $N = 5000$, damage fraction $f = 0.3$.

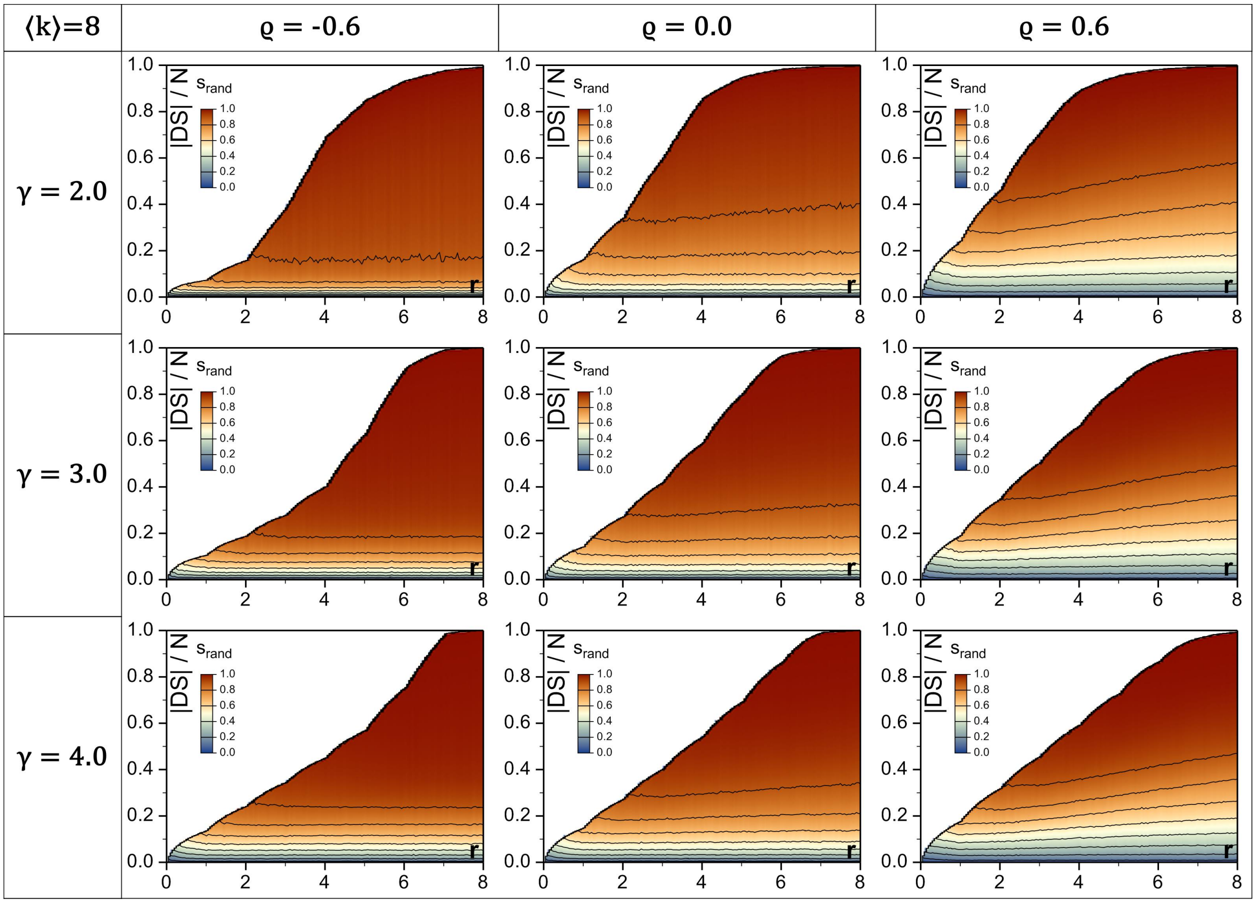

Supplementary Figure S13: Stability of partial frDS against random damage, as a function of redundancy level and dominating set size, at various power-law degree exponents and Spearman's ρ values. Synthetic networks, $N = 5000$, damage fraction $f = 0.5$.

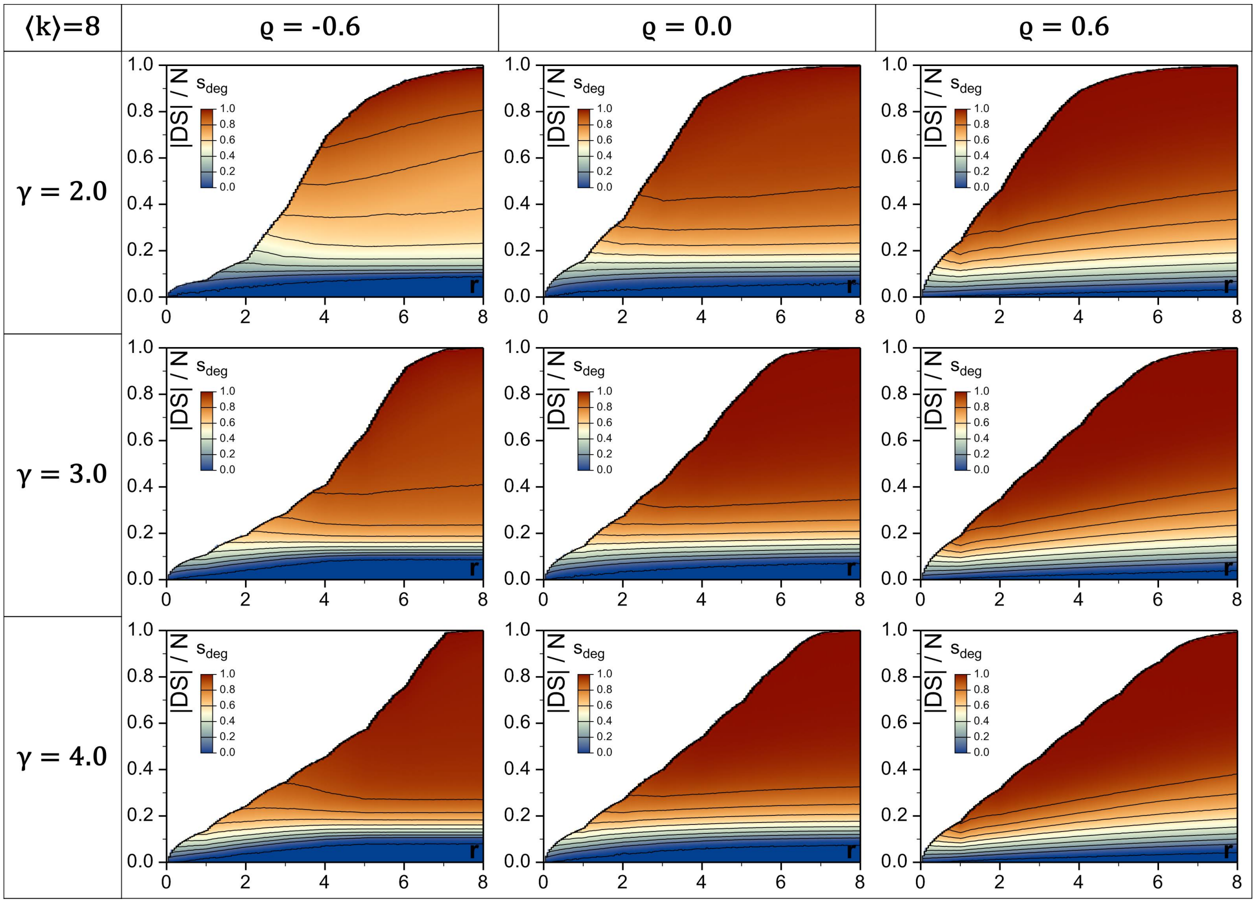

Supplementary Figure S14: Stability of partial frDS against targeted attack, as a function of redundancy level and dominating set size, at various power-law degree exponents and Spearman's ρ values. Synthetic networks, $N = 5000$, damage fraction $f = 0.1$.

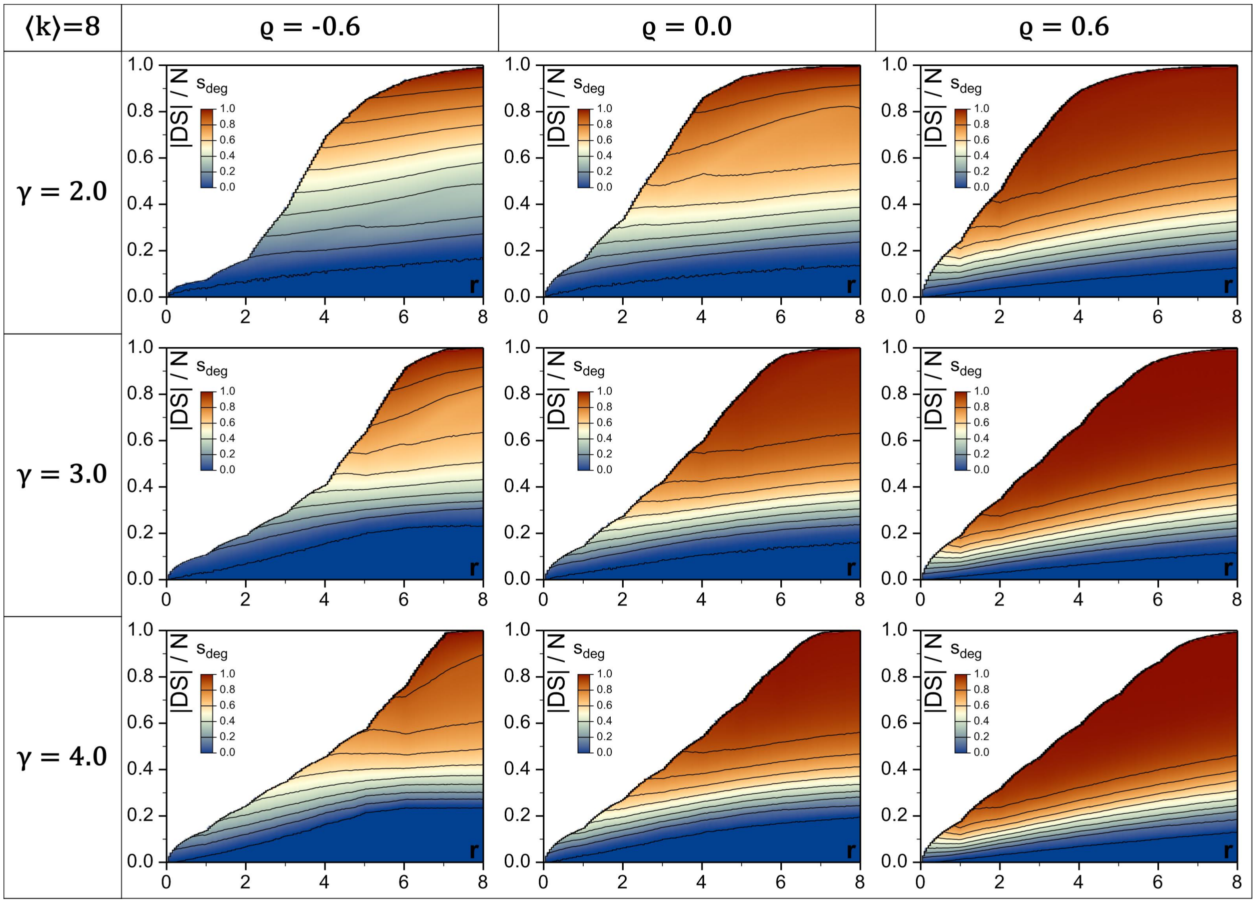

Supplementary Figure S15: Stability of partial frDS against targeted attack, as a function of redundancy level and dominating set size, at various power-law degree exponents and Spearman's ρ values. Synthetic networks, $N = 5000$, damage fraction $f = 0.3$.

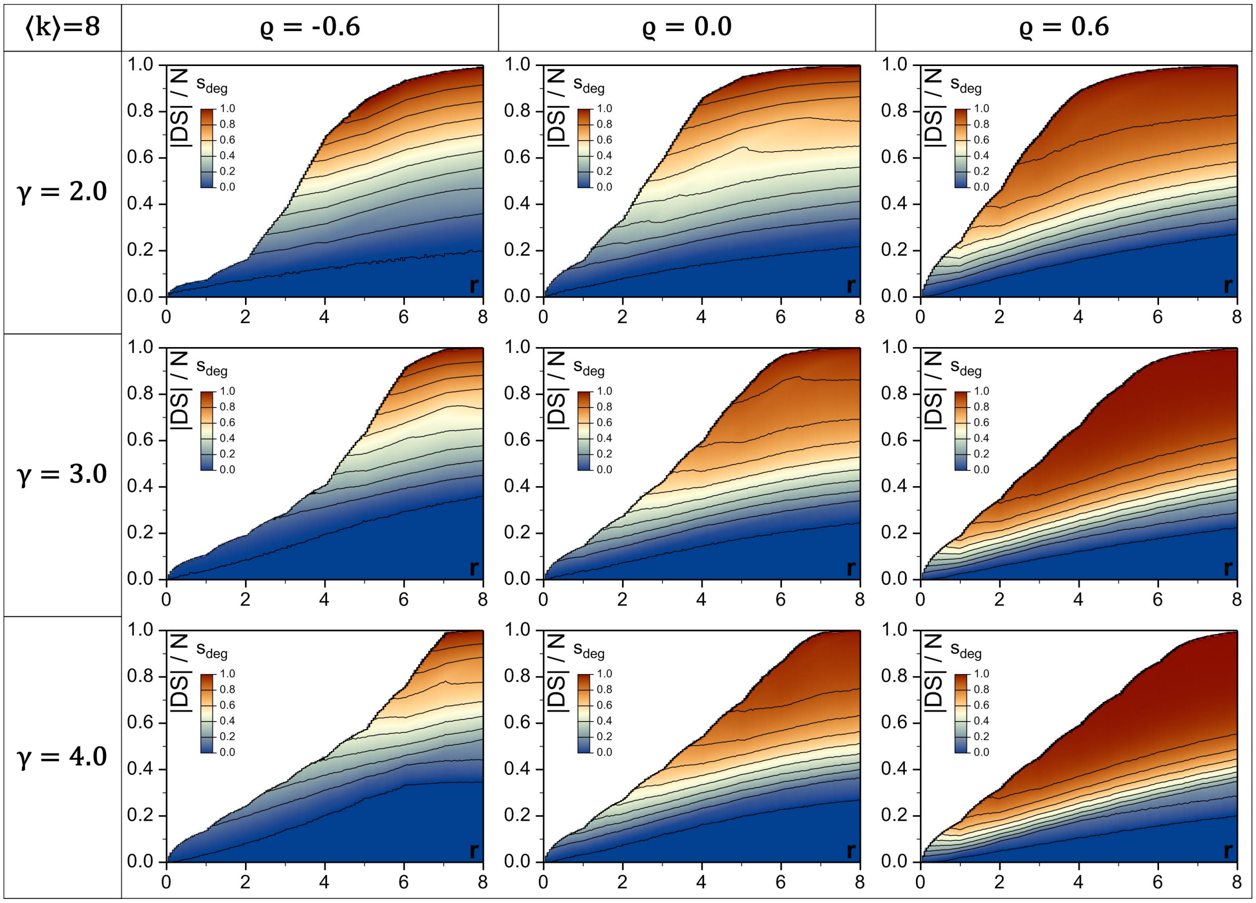

Supplementary Figure S16: Stability of partial frDS against targeted attack, as a function of redundancy level and dominating set size, at various power-law degree exponents and Spearman's ρ values. Synthetic networks, $N = 5000$, damage fraction $f = 0.5$.

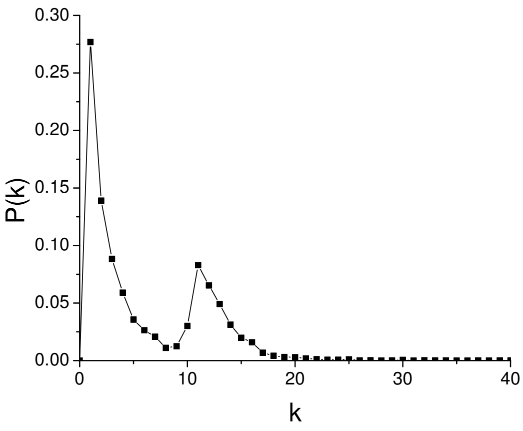

Supplementary Figure S17: Degree distribution of Gnutella08 network [7] on linear scale.

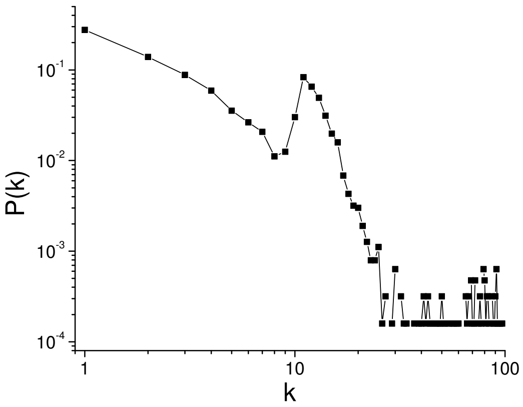

Supplementary Figure S18: Degree distribution of Gnutella08 network [7] on double-logarithmic scale.

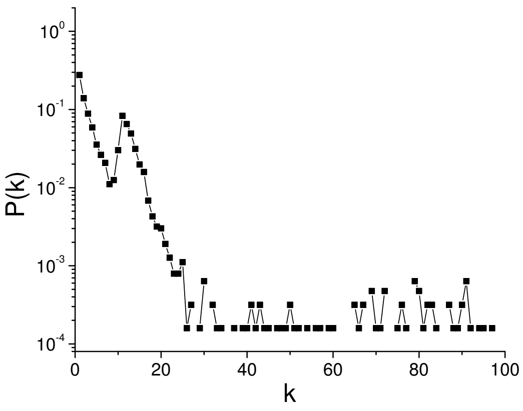

Supplementary Figure S19: Degree distribution of Gnutella08 network [7] on log-linear scale.

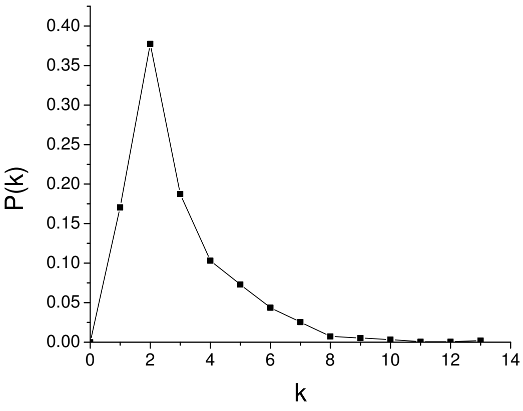

Supplementary Figure S20: Degree distribution of ENTSO-E powergrid [8, 9] on linear scale.

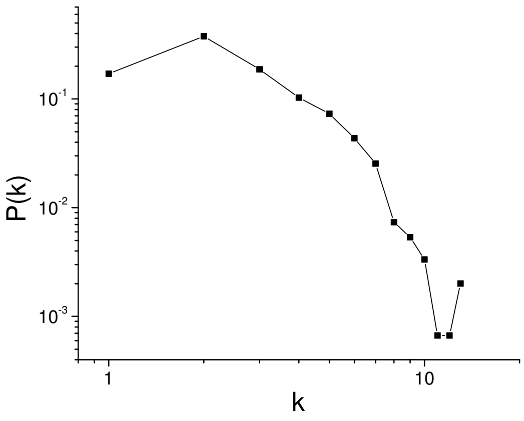

Supplementary Figure S21: Degree distribution of ENTSO-E powergrid [8, 9] on double-logarithmic scale.

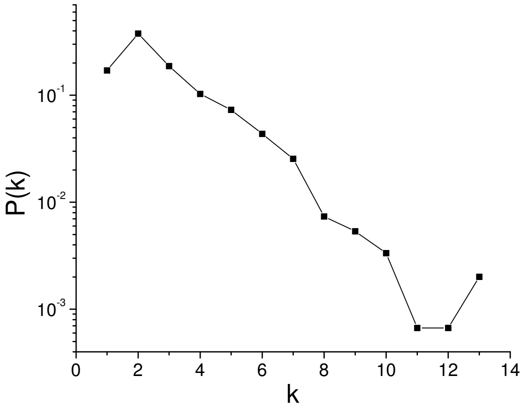

Supplementary Figure S22: Degree distribution of ENTSO-E powergrid [8, 9] on log-linear scale.

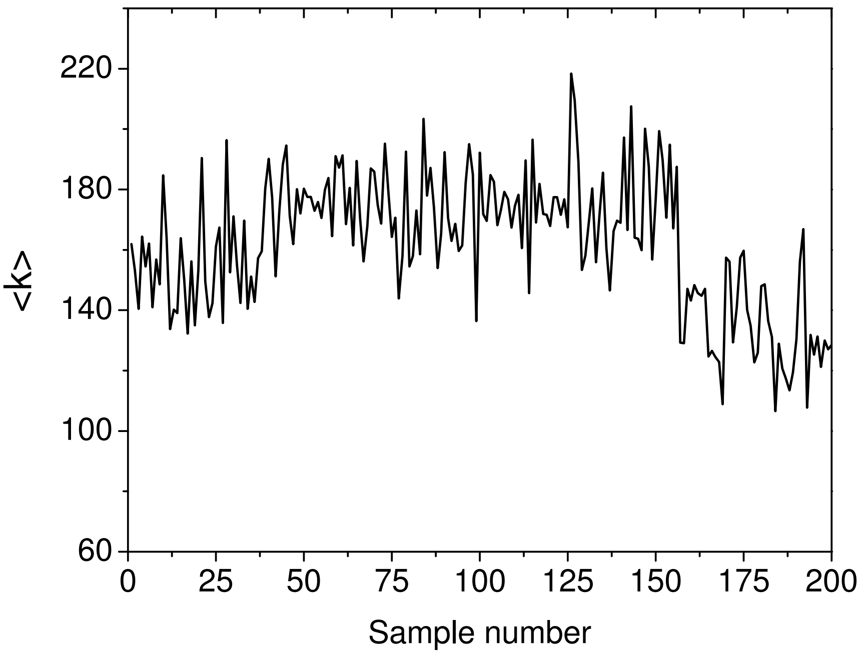

Supplementary Figure S23: Average degree in brain graphs. See Supplementary Table S1 for sample numbers.

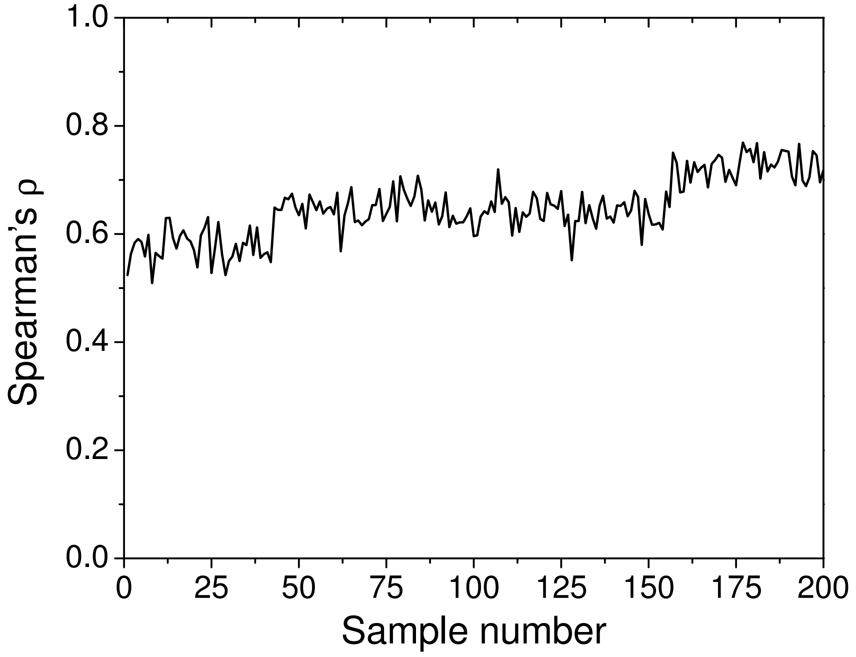

Supplementary Figure S24: Assortativity of brain graphs. See Supplementary Table S1 for sample numbers.

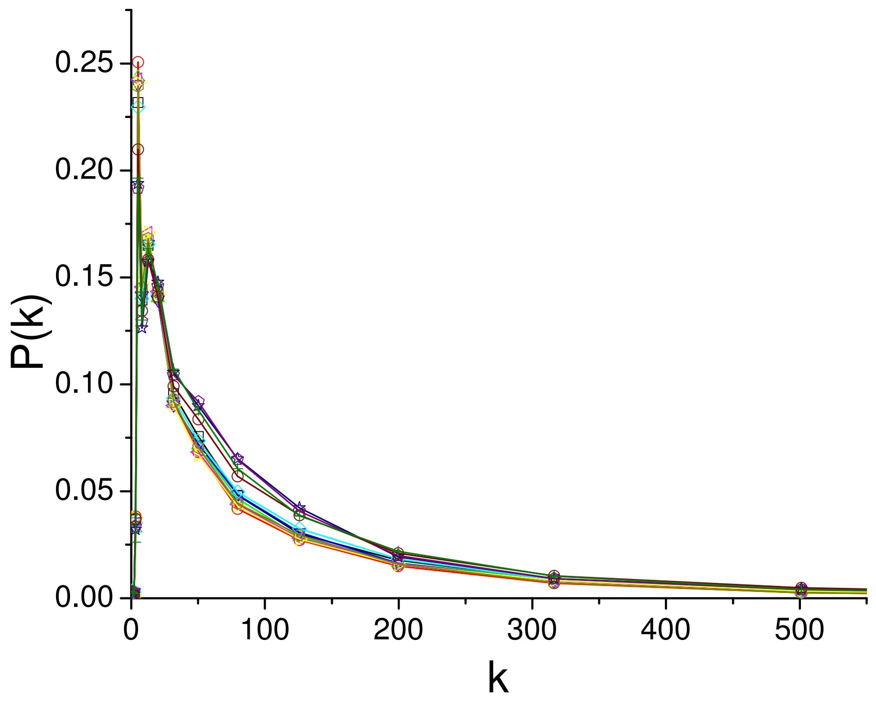

Supplementary Figure S25: Degree distributions of 12 randomly picked brain graphs on linear scale.

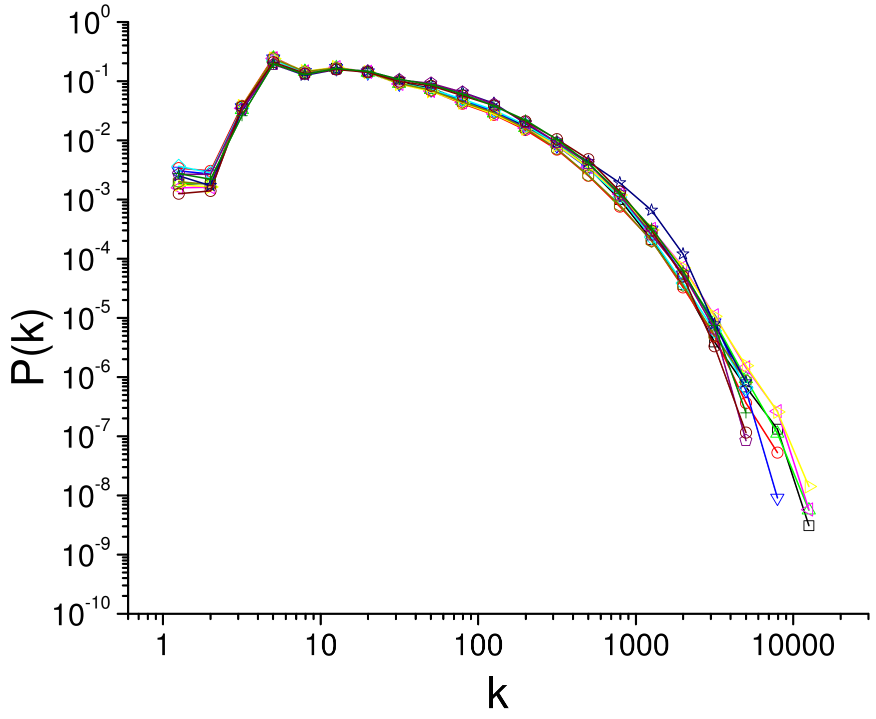

Supplementary Figure S26: Degree distributions of 12 randomly picked brain graphs on double-logarithmic scale.

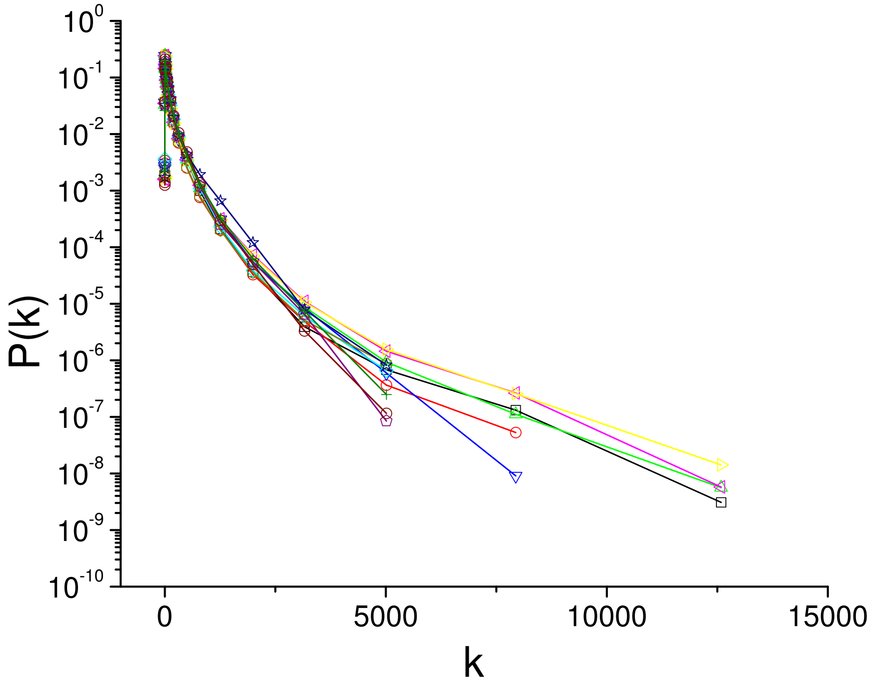

Supplementary Figure S27: Degree distributions of 12 randomly picked brain graphs on log-linear scale.

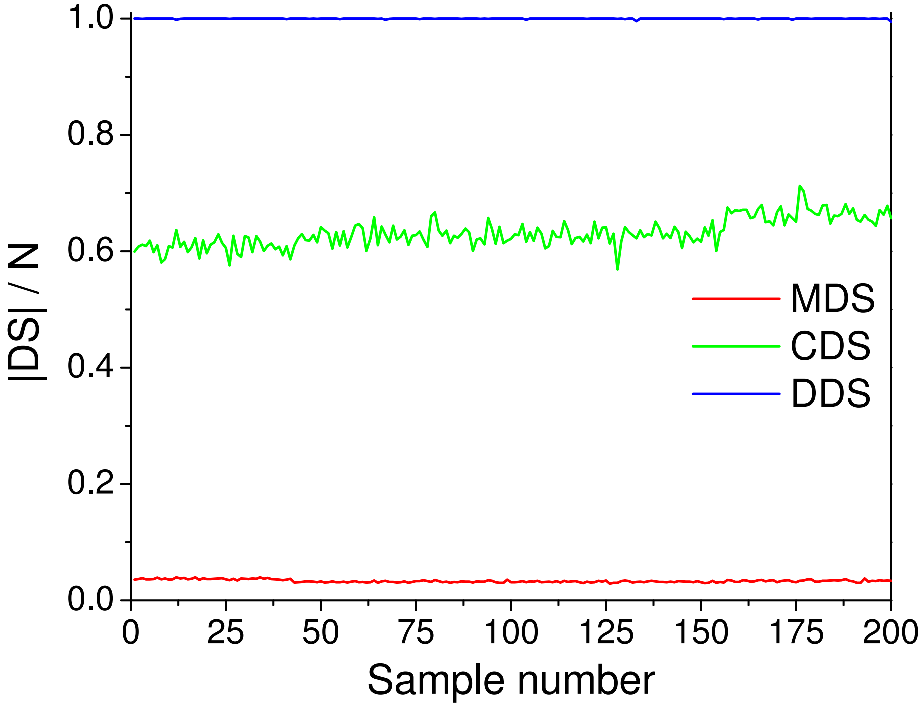

Supplementary Figure S28: Comparison of dominating set sizes in brain graphs. See Supplementary Table S1 for sample numbers.

sample#	graph	sample#	graph
1	KKI-21_KKI2009-01_big_graph_w_inv	51	MRN114_M87114047_big_graph_w_inv
2	KKI-21_KKI2009-02_big_graph_w_inv	52	MRN114_M87114064_big_graph_w_inv
3	KKI-21_KKI2009-03_big_graph_w_inv	53	MRN114_M87115498_big_graph_w_inv
4	KKI-21_KKI2009-04_big_graph_w_inv	54	MRN114_M87115517_big_graph_w_inv
5	KKI-21_KKI2009-05_big_graph_w_inv	55	MRN114_M87117119_big_graph_w_inv
6	KKI-21_KKI2009-06_big_graph_w_inv	56	MRN114_M87117167_big_graph_w_inv
7	KKI-21_KKI2009-07_big_graph_w_inv	57	MRN114_M87120962_big_graph_w_inv
8	KKI-21_KKI2009-08_big_graph_w_inv	58	MRN114_M87121943_big_graph_w_inv
9	KKI-21_KKI2009-09_big_graph_w_inv	59	MRN114_M87121956_big_graph_w_inv
10	KKI-21_KKI2009-10_big_graph_w_inv	60	MRN114_M87122092_big_graph_w_inv
11	KKI-21_KKI2009-11_big_graph_w_inv	61	MRN114_M87123042_big_graph_w_inv
12	KKI-21_KKI2009-12_big_graph_w_inv	62	MRN114_M87123449_big_graph_w_inv
13	KKI-21_KKI2009-13_big_graph_w_inv	63	MRN114_M87123913_big_graph_w_inv
14	KKI-21_KKI2009-14_big_graph_w_inv	64	MRN114_M87124633_big_graph_w_inv
15	KKI-21_KKI2009-15_big_graph_w_inv	65	MRN114_M87124781_big_graph_w_inv
16	KKI-21_KKI2009-16_big_graph_w_inv	66	MRN114_M87124827_big_graph_w_inv
17	KKI-21_KKI2009-17_big_graph_w_inv	67	MRN114_M87125134_big_graph_w_inv
18	KKI-21_KKI2009-18_big_graph_w_inv	68	MRN114_M87128444_big_graph_w_inv
19	KKI-21_KKI2009-19_big_graph_w_inv	69	MRN114_M87129719_big_graph_w_inv
20	KKI-21_KKI2009-20_big_graph_w_inv	70	MRN114_M87129789_big_graph_w_inv
21	KKI-21_KKI2009-21_big_graph_w_inv	71	MRN114_M87131806_big_graph_w_inv
22	KKI-21_KKI2009-22_big_graph_w_inv	72	MRN114_M87134068_big_graph_w_inv
23	KKI-21_KKI2009-23_big_graph_w_inv	73	MRN114_M87135647_big_graph_w_inv
24	KKI-21_KKI2009-24_big_graph_w_inv	74	MRN114_M87136332_big_graph_w_inv
25	KKI-21_KKI2009-25_big_graph_w_inv	75	MRN114_M87136832_big_graph_w_inv
26	KKI-21_KKI2009-26_big_graph_w_inv	76	MRN114_M87139021_big_graph_w_inv
27	KKI-21_KKI2009-27_big_graph_w_inv	77	MRN114_M87139257_big_graph_w_inv
28	KKI-21_KKI2009-28_big_graph_w_inv	78	MRN114_M87141220_big_graph_w_inv
29	KKI-21_KKI2009-29_big_graph_w_inv	79	MRN114_M87141664_big_graph_w_inv
30	KKI-21_KKI2009-30_big_graph_w_inv	80	MRN114_M87141793_big_graph_w_inv
31	KKI-21_KKI2009-31_big_graph_w_inv	81	MRN114_M87141858_big_graph_w_inv
32	KKI-21_KKI2009-32_big_graph_w_inv	82	MRN114_M87141906_big_graph_w_inv
33	KKI-21_KKI2009-33_big_graph_w_inv	83	MRN114_M87141949_big_graph_w_inv
34	KKI-21_KKI2009-34_big_graph_w_inv	84	MRN114_M87142764_big_graph_w_inv
35	KKI-21_KKI2009-35_big_graph_w_inv	85	MRN114_M87143273_big_graph_w_inv
36	KKI-21_KKI2009-36_big_graph_w_inv	86	MRN114_M87144889_big_graph_w_inv
37	KKI-21_KKI2009-37_big_graph_w_inv	87	MRN114_M87144896_big_graph_w_inv
38	KKI-21_KKI2009-38_big_graph_w_inv	88	MRN114_M87145479_big_graph_w_inv
39	KKI-21_KKI2009-39_big_graph_w_inv	89	MRN114_M87145575_big_graph_w_inv
40	KKI-21_KKI2009-40_big_graph_w_inv	90	MRN114_M87146520_big_graph_w_inv
41	KKI-21_KKI2009-41_big_graph_w_inv	91	MRN114_M87146993_big_graph_w_inv
42	KKI-21_KKI2009-42_big_graph_w_inv	92	MRN114_M87147006_big_graph_w_inv
43	MRN114_M87102217_big_graph_w_inv	93	MRN114_M87148745_big_graph_w_inv
44	MRN114_M87102806_big_graph_w_inv	94	MRN114_M87149014_big_graph_w_inv
45	MRN114_M87103074_big_graph_w_inv	95	MRN114_M87149025_big_graph_w_inv
46	MRN114_M87105476_big_graph_w_inv	96	MRN114_M87150194_big_graph_w_inv
47	MRN114_M87107085_big_graph_w_inv	97	MRN114_M87150415_big_graph_w_inv
48	MRN114_M87108094_big_graph_w_inv	98	MRN114_M87150639_big_graph_w_inv
49	MRN114_M87111487_big_graph_w_inv	99	MRN114_M87151117_big_graph_w_inv
50	MRN114_M87111924_big_graph_w_inv	100	MRN114_M87151146_big_graph_w_inv

sample#	graph	sample#	graph
101	MRN114_M87151453_big_graph_w_inv	151	MRN114_M87192995_big_graph_w_inv
102	MRN114_M87152844_big_graph_w_inv	152	MRN114_M87193409_big_graph_w_inv
103	MRN114_M87153569_big_graph_w_inv	153	MRN114_M87196363_big_graph_w_inv
104	MRN114_M87154559_big_graph_w_inv	154	MRN114_M87196591_big_graph_w_inv
105	MRN114_M87155496_big_graph_w_inv	155	MRN114_M87199297_big_graph_w_inv
106	MRN114_M87155949_big_graph_w_inv	156	MRN114_M87199728_big_graph_w_inv
107	MRN114_M87156106_big_graph_w_inv	157	NKI-TRT_0021001_1_big_graph_w_inv
108	MRN114_M87157827_big_graph_w_inv	158	NKI-TRT_0021001_2_big_graph_w_inv
109	MRN114_M87158338_big_graph_w_inv	159	NKI-TRT_0021002_1_big_graph_w_inv
110	MRN114_M87158534_big_graph_w_inv	160	NKI-TRT_0021002_2_big_graph_w_inv
111	MRN114_M87159410_big_graph_w_inv	161	NKI-TRT_0021006_1_big_graph_w_inv
112	MRN114_M87159580_big_graph_w_inv	162	NKI-TRT_0021006_2_big_graph_w_inv
113	MRN114_M87160332_big_graph_w_inv	163	NKI-TRT_0021018_1_big_graph_w_inv
114	MRN114_M87160375_big_graph_w_inv	164	NKI-TRT_0021018_2_big_graph_w_inv
115	MRN114_M87161235_big_graph_w_inv	165	NKI-TRT_0021024_1_big_graph_w_inv
116	MRN114_M87161902_big_graph_w_inv	166	NKI-TRT_0021024_2_big_graph_w_inv
117	MRN114_M87162915_big_graph_w_inv	167	NKI-TRT_1427581_2_big_graph_w_inv
118	MRN114_M87164412_big_graph_w_inv	168	NKI-TRT_1793622_1_big_graph_w_inv
119	MRN114_M87164886_big_graph_w_inv	169	NKI-TRT_1793622_2_big_graph_w_inv
120	MRN114_M87165017_big_graph_w_inv	170	NKI-TRT_1961098_1_big_graph_w_inv
121	MRN114_M87165441_big_graph_w_inv	171	NKI-TRT_1961098_2_big_graph_w_inv
122	MRN114_M87166115_big_graph_w_inv	172	NKI-TRT_2475376_1_big_graph_w_inv
123	MRN114_M87168759_big_graph_w_inv	173	NKI-TRT_2475376_2_big_graph_w_inv
124	MRN114_M87174803_big_graph_w_inv	174	NKI-TRT_2799329_1_big_graph_w_inv
125	MRN114_M87176019_big_graph_w_inv	175	NKI-TRT_2799329_2_big_graph_w_inv
126	MRN114_M87176708_big_graph_w_inv	176	NKI-TRT_2842950_1_big_graph_w_inv
127	MRN114_M87178630_big_graph_w_inv	177	NKI-TRT_2842950_2_big_graph_w_inv
128	MRN114_M87179511_big_graph_w_inv	178	NKI-TRT_3201815_1_big_graph_w_inv
129	MRN114_M87179597_big_graph_w_inv	179	NKI-TRT_3201815_2_big_graph_w_inv
130	MRN114_M87179713_big_graph_w_inv	180	NKI-TRT_3313349_1_big_graph_w_inv
131	MRN114_M87181205_big_graph_w_inv	181	NKI-TRT_3313349_2_big_graph_w_inv
132	MRN114_M87181216_big_graph_w_inv	182	NKI-TRT_3315657_1_big_graph_w_inv
133	MRN114_M87182922_big_graph_w_inv	183	NKI-TRT_3315657_2_big_graph_w_inv
134	MRN114_M87183189_big_graph_w_inv	184	NKI-TRT_3795193_1_big_graph_w_inv
135	MRN114_M87183485_big_graph_w_inv	185	NKI-TRT_3795193_2_big_graph_w_inv
136	MRN114_M87184910_big_graph_w_inv	186	NKI-TRT_3808535_1_big_graph_w_inv
137	MRN114_M87185000_big_graph_w_inv	187	NKI-TRT_3808535_2_big_graph_w_inv
138	MRN114_M87186642_big_graph_w_inv	188	NKI-TRT_3893245_2_big_graph_w_inv
139	MRN114_M87187090_big_graph_w_inv	189	NKI-TRT_4176156_1_big_graph_w_inv
140	MRN114_M87187750_big_graph_w_inv	190	NKI-TRT_4176156_2_big_graph_w_inv
141	MRN114_M87187984_big_graph_w_inv	191	NKI-TRT_4288245_1_big_graph_w_inv
142	MRN114_M87188000_big_graph_w_inv	192	NKI-TRT_4288245_2_big_graph_w_inv
143	MRN114_M87188762_big_graph_w_inv	193	NKI-TRT_6471972_1_big_graph_w_inv
144	MRN114_M87190609_big_graph_w_inv	194	NKI-TRT_7055197_1_big_graph_w_inv
145	MRN114_M87190745_big_graph_w_inv	195	NKI-TRT_7055197_2_big_graph_w_inv
146	MRN114_M87191087_big_graph_w_inv	196	NKI-TRT_8574662_1_big_graph_w_inv
147	MRN114_M87191258_big_graph_w_inv	197	NKI-TRT_8735778_1_big_graph_w_inv
148	MRN114_M87192333_big_graph_w_inv	198	NKI-TRT_8735778_2_big_graph_w_inv
149	MRN114_M87192557_big_graph_w_inv	199	NKI-TRT_9630905_1_big_graph_w_inv
150	MRN114_M87192637_big_graph_w_inv	200	NKI-TRT_9630905_2_big_graph_w_inv

Supplementary Table S1: Sample numbers of brain graphs that we use to identify them in our figures. Graph data is available with identical file names at [10].

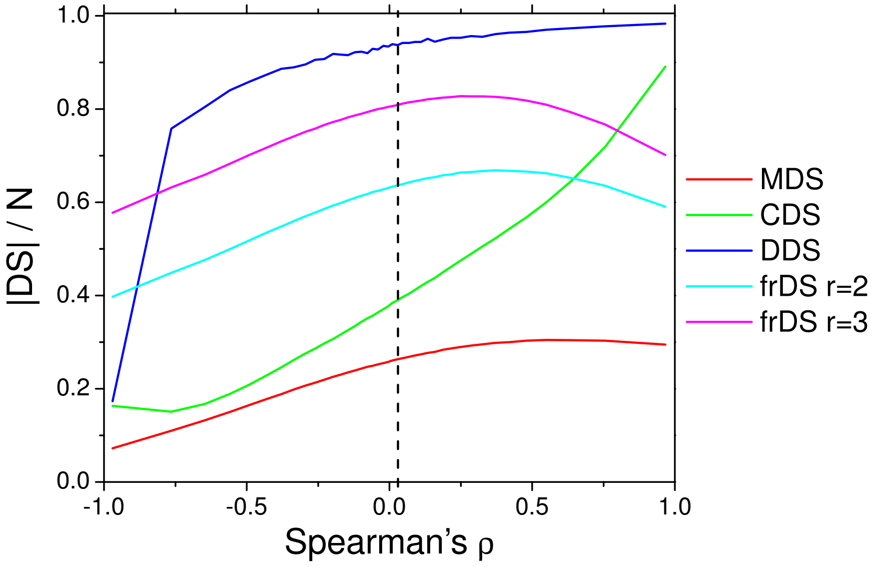

Supplementary Figure S29: Dominating set sizes vs. assortativity in **Gnutella08** graph [7], achieved by random (biased) mixing of exges by double-edge swaps. The vertical dashed line indicates the assortativity of the original graph.

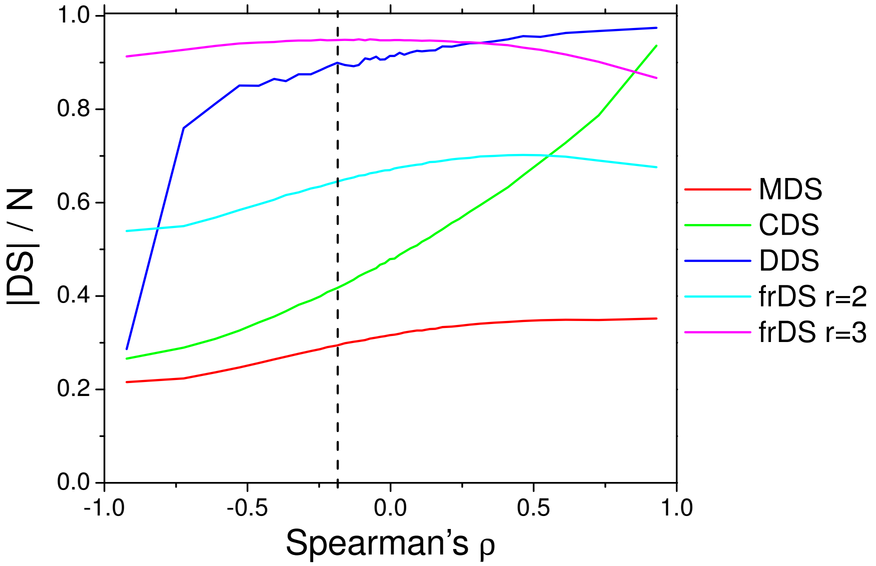

Supplementary Figure S30: Dominating set sizes vs. assortativity in **powergrid** graph [8, 9], achieved by random (biased) mixing of exges by double-edge swaps. The vertical dashed line indicates the assortativity of the original graph.

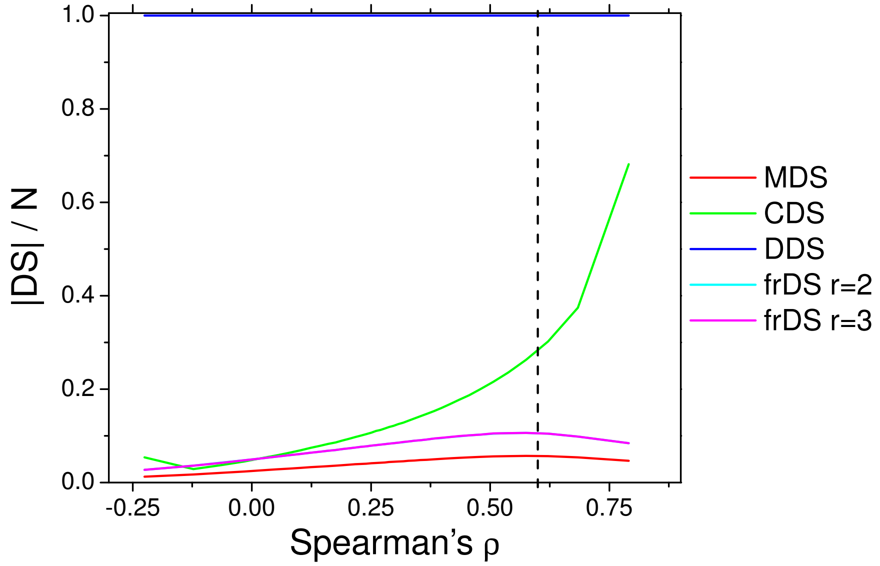

Supplementary Figure S31: Dominating set sizes vs. assortativity in brain graph **KKI-21_KKI2009-19** [10, 11], achieved by random (biased) mixing of edges by double-edge swaps. The vertical dashed line indicates the assortativity of the original graph. Note that frDS curves with $r = 2$ and $r = 3$ overlap.

Supplementary References

[1] Spearman, C. The Proof and Measurement of Association between Two Things. *The American Journal of Psychology* **15**, 72–101 (1904).

[2] Litvak, N. and van der Hofstad, R. Uncovering disassortativity in large scale-free networks. *Phys. Rev. E* **87**, 022801 (2013).

[3] Newman, M. Mixing patterns in networks. *Phys. Rev. E* **67**, 026126 (2003).

[4] Newman, M. Assortative mixing in networks. *Phys. Rev. Lett.* **89**, 208701 (2002).

[5] Molnár, F. Jr. et al. Dominating Scale-Free Networks Using Generalized Probabilistic Methods. *Sci. Rep.* **4,** 6308 (2014).

[6] Viger, F., Latapy, M. Efficient and simple generation of random simple connected graphs with prescribed degree sequence. In Proc. The 11th Intl. Comp. and Combin. Conf. 440–449 (2005).

[7] Stanford Network Analysis Project (SNAP), http://snap.stanford.edu/data, Accessed 02/12/2013.

[8] Hutcheon, N., Bialek, J.W. Updated and validated power flow model of the main continental European transmission network. *PowerTech, IEEE Grenoble*, 16–20 (2013).

[9] Continental European Transmission Network (2009 winter data), http://www.powerworld.com/bialek, Accessed 01/08/2014.

[10] Open Connectome Project, http://mrbrain.cs.jhu.edu/disa/download, Accessed 03/07/2014.

[11] Roncal, W.G. et al. MIGRAINE: MRI Graph Reliability Analysis and Inference for Connectomics. *GlobalSIP, IEEE*, 313–316 (2013).
